# Structural and enzymatic insights into QatD, a dual-function TatD-like nuclease in the QatABCD anti-phage defense system

**DOI:** 10.1093/nar/gkag776

**Published:** 2026-08-04

**Authors:** Xu Wang, Na Wang, Lin Zhang, Meilin Zhang, Yaling Xu, Zheng Cao, Honghua Ge, Jinming Ma

**Affiliations:** School of Life Sciences and Medical Engineering, Anhui University, Hefei 230601, China; School of Life Sciences and Medical Engineering, Anhui University, Hefei 230601, China; Beijing Obstetrics and Gynecology Hospital, Capital Medical University, Beijing Maternal and Child Health Care Hospital, No.251 Yaojiayuan Rd, Beijing 100026, China; School of Life Sciences and Medical Engineering, Anhui University, Hefei 230601, China; School of Life Sciences and Medical Engineering, Anhui University, Hefei 230601, China; Beijing Obstetrics and Gynecology Hospital, Capital Medical University, Beijing Maternal and Child Health Care Hospital, No.251 Yaojiayuan Rd, Beijing 100026, China; School of Life Sciences and Medical Engineering, Anhui University, Hefei 230601, China; School of Life Sciences and Medical Engineering, Anhui University, Hefei 230601, China

## Abstract

The *qatABCD* system is a widespread anti-phage module featuring a core QatBC complex, but the specific biological role of its conserved component, QatD, has long been enigmatic. Here, we establish QatD is a TatD-family nuclease co-opted for antiviral defense. The crystal structure of *Acinetobacter baumannii* QatD reveals a classic TIM-barrel fold featuring a conserved “HxH” active-site motif characteristic of Type II TatD enzymes. Biochemically, QatD exhibits metal-dependent dual activity: a Mg^2+^-dependent 3′-5′ exonuclease and a Ca^2+^-dependent apurinic/apyrimidinic (AP) endonuclease. We further demonstrate that QatD confers resistance against diverse bacteriophages *in vivo*, suggesting its defense function is tied to its catalytic activity. Crucially, we discovered that the nuclease activity of QatD is tightly inhibited by physiological concentrations of host nucleoside triphosphates (NTPs). Based on these findings, we propose a mechanistic model wherein the massive nucleotide consumption during rapid viral transcription and replication depletes local host NTP pools, thereby relieving the metabolic inhibition on QatD. The unleashed QatD subsequently targets and degrades single-stranded replication intermediates and AP-site-containing viral genomes. Our work not only elucidates the molecular basis of QatD activation but also highlights an elegant evolutionary strategy wherein bacteria couple metabolic sensing with ancient DNA repair machinery for specialized immune defense.

## Graphical Abstract

**Graphical Abstract:**
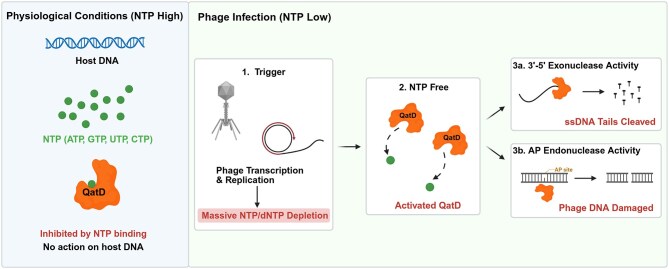

## Introduction

Bacteria and archaea deploy an extraordinary variety of molecular systems to defend themselves against bacteriophage infection [1–4]. Beyond canonical restriction–modification, and CRISPR–Cas pathways, large-scale genomic and functional screens have uncovered a vast repertoire of previously unrecognized anti-phage operons, many encoding nucleases or metabolic enzymes co-opted for immune defense [5–10]. These systems often repurpose ancient enzyme folds to detect and destroy viral nucleic acids, forming a dynamic arms race between prokaryotes and their phages. For example, Gao *et al*. systematically identified 29 new widespread defense gene cassettes (in ~32% of sequenced microbial genomes) with functions including RNA editing, novel nucleases, and small-molecule modifiers [7]. One such locus, designated *qatABCD*, was predicted to encode a multi-protein defense system [11]. The *qatABCD* operon comprises four genes: *qatA*, a member of the KAP-family ATPase/NTPase; *qatB*, a small protein of unknown fold; *qatC*, a homolog of the QueC enzyme involved in 7-deazaguanine (queuosine) biosynthesis; and *qatD*, a predicted TatD-family nuclease [11–13].

Recently, Gao *et al*. [14], Oh *et al*. [15], and Gao *et al*. [16] demonstrated that QatB and QatC form a stable heterocomplex in which the N-terminal domain of QatB inserts into the catalytic pocket of QatC—an interaction pattern highly reminiscent of CdnD insertion into the Cap9 catalytic center [17]. The QatBC complex alone is sufficient to confer protection against a range of double-stranded DNA (dsDNA) phages in *Escherichia coli*, suggesting it functions as the core antiviral effector complex [14]. Notably, however, QatD is conserved in essentially all known *qat* operons across diverse bacteria, suggesting an important yet cryptic role.

QatD belongs to the classical TatD family, which have been characterized as metal dependent nucleases [18–23]. Originally annotated in the *E. coli* twin-arginine translocation (Tat) operon, TatD was later shown to function independently as a Mg^2+^-dependent DNase [18, 24]. Biochemical and structural work has since established that TatD enzymes possess dual 3′-5′ exonuclease and apurinic/apyrimidinic (AP) endonuclease activities [25]. These activities are supported by a conserved (G/A/S)xN or HxH catalytic motif that coordinates two divalent metal ions—typically Mg^2+^, Mn^2+^, or Ca^2+^—to promote phosphodiester hydrolysis via a two-metal-ion mechanism. The discovery that human and bacterial TatD orthologs share this bifunctional nuclease behavior underscores their ancient evolutionary origin and biochemical versatility [25]. Functional diversity within the TatD family extends beyond nucleic acid repair to roles in host-pathogen conflict. For example, the *Plasmodium falciparum* TatD-like DNase degrades neutrophil extracellular traps, thereby promoting parasite virulence, while deletion of tatD homologs in bacteria sensitizes cells to oxidative stress and DNA damage [26]. Such findings raise the possibility that TatD nucleases can be co-opted as molecular weapons in prokaryotic immune systems.

Indeed, Wang *et al*. systematically mapped nuclease-containing defense systems in *E. coli* and identified QatABCD among a subset of operons encoding multi-component nuclease-based phage defense modules [27]. Their functional assays demonstrated that QatD enhances the resistance spectrum and robustness of the QatABCD system. Taken together, these findings position QatD as a representative of an emerging class of TatD-derived immune nucleases, potentially bridging DNA repair and antiviral defense. Despite its evolutionary conservation and predicted catalytic potential, the precise biochemical function of QatD and its molecular interplay with QatBC remain unknown. Here, we combine structural, biochemical, and functional analyses to elucidate the role of QatD in the QatABCD system. We determined its crystal structure, defined its metal cofactor and nucleic acid substrate preferences, and preliminarily evaluated how catalytic mutations affect phage resistance. By establishing the mechanistic basis of QatD activity, our study provides insights into how bacteria have adapted an ancient DNA repair enzyme into a phage defense factor, expanding the repertoire of nuclease-based immunity in prokaryotes.

## Materials and methods

### Plasmid construction

The *qatD* gene was amplified from the genomic DNA of an anti-phage *Acinetobacter baumannii* strain using PCR (P528, Vazyme Biotech Co., Ltd) and subsequently cloned into a modified pET22b (+) expression vector (Novagen) with a C-terminal 6 × His tag. All mutations used in this study were introduced using standard PCR procedures. Plasmids used in this study are listed in Supplementary Table S1. All plasmids used in this study were sequenced-confirmed.

### Protein expression and purification

The verified plasmid was transformed into *E. coli* BL21 (DE3) via heat shock (EC1002, Shanghai Weidi). Cells were grown at 37°C in Luria-Bertani (LB) medium supplemented with ampicillin (50 μg/ml). Protein expression was induced with 0.2 mM isopropyl β-d-thiogalactoside (IPTG) when the OD_600_ reached 0.6, and the cells were harvested after overnight incubation at 16°C and 180 rpm. The collected cell pellet was resuspended in lysis buffer (500 mM NaCl, 20 mM HEPES, pH 8.0). The resuspended cells were lysed by sonication followed by centrifugation at 13 680 × *g* for 1 h. The supernatant containing soluble QatD protein was incubated with Ni-Sepharose affinity beads (GE Healthcare) and washed with wash buffer (20 mM imidazole, 500 mM NaCl, 20 mM HEPES, pH 8.0). Recombinant QatD protein was eluted with elute buffer (200 mM imidazole, 500 mM NaCl, 20 mM HEPES, pH 8.0), the protein was further purified using a Superdex 200 column (GE Healthcare) with equilibration buffer (150 mM NaCl, 20 mM HEPES, pH 8.0). Purified protein samples were rapidly frozen in liquid nitrogen and stored at −80°C for future use.

### Crystallization, data collection, and structure determination

The protein samples used for crystallization were mixed with precipitant [0.15 M DL-Malic acid, pH 7.0, 20% (w/v) Polyethylene glycol 3350] in a 1:1 ratio at concentrations of 6 mg/ml. The crystallization experiments were conducted using the sitting drop method at 16°C. All crystals were immersed in cryoprotectants derived from the mother liquors supplemented with 15%–30% glycerol before rapid freezing in liquid nitrogen.

The diffraction data for all crystals were collected at a wavelength of 0.9793 Å at beamline 18U (BL18U1) at Shanghai Synchrotron Radiation Facility (SSRF), China. Following processing with AutoPX [28], the QatD structure was solved through molecular replacement in PHASER [29] using the AlphaFold model as the search model and subsequently manually refined and built using Coot [30]. All structure was refined by Phenix.refine [31]. Supplementary Table S2 summarizes the statistics for data collection and structure refinement. All Figures were prepared using PyMOL (The Pymol Molecular Graphics System, Schrödinger).

### *In silico* QatD–DNA docking

To gain further insight into the DNA-binding mode of QatD, we employed RoseTTAFold All-Atom to *de novo* generate structural models of QatD in complex with DNA [32]. A 25 bp DNA duplex (5′-CGTCTCCTGAACTGTGCTATCGCTC-3′) was provided as the nucleic-acid component for co-modeling. The resulting QatD–DNA models were subsequently evaluated against the solved crystal structure of QatD to assess stereochemical consistency and to confirm that the predicted DNA-contacting residues are compatible with the experimentally observed active-site and channel architecture.

### Nuclease activity assay

The experimental procedure was performed as described previously by Dorival *et al*. [25], with minor modifications. All oligonucleotides used were synthesized by GENERAL BIOL, and their sequences are provided in Supplementary Table S3. To generate DNA substrates containing natural AP sites, dU-containing oligonucleotides were treated with uracil–DNA glycosylase (UDG, Beyotime) according to the manufacturer’s instructions. Standard enzyme digestion reactions were carried out at 37°C for indicated durations. Unless specified otherwise, the same amounts of 0.5 μM protein and 0.1 μM DNA substrates were maintained across parallel groups (5:1 protein-to-DNA molar ratio). To examine metal ion dependencies or support specific cleavage activities throughout this study, the reaction buffers were supplemented with various divalent cations at the following fixed final concentrations: 10 mM CuCl_2_, 0.5 mM ZnCl_2_, 3 mM MnCl_2_, 10 mM MgCl_2_, 5 mM CaCl_2_, 1 mM NiCl_2_, 5 mM CoCl_2_ or 10 mM ethylenediaminetetraacetic acid (EDTA) (as a metal-free control). For NTP/dNTP inhibition assays, reactions were supplemented with a range of concentrations [0–10 mM for nucleoside triphosphates (NTPs) and 0–2 mM for dNTPs] of the indicated nucleotides, while keeping all other reaction components unchanged. Reactions intended for agarose gel electrophoresis (AG0100, Beijing LABLEAD Inc.) were terminated by the addition of 10 mM EDTA. Conversely, reactions designated for denaturing 20% polyacrylamide gel electrophoresis (PAGE) containing 7 M urea were stopped using a loading buffer consisting of 96% formamide, 10 mM EDTA, and 2 mg/ml Orange G [33, 34]. Finally, the reaction products were resolved by corresponding gel electrophoresis, and all images of FAM-labeled DNA were captured using a Typhoon 9500 imaging system (GE Healthcare). Unless otherwise stated, qualitative gel electrophoresis assays were performed as single independent experiments to confirm the presence or distinct visual features of the bands.

### Phylogenetic analysis and residue conservation prediction of QatD

Protein sequences of QatD were retrieved from the NCBI Protein Database (https://www.ncbi.nlm.nih.gov/protein/). Only sequences associated with genomic accession numbers were selected, and their amino acid sequences were downloaded in FASTA format. Multiple sequence alignment of the QatD protein sequences was performed using the ClustalW algorithm with default parameters in MEGA software [35]. Phylogenetic tree construction was carried out using the neighbor-joining method in MEGA. The parameters for tree construction were set as follows: phylogenetic test was the Bootstrap method with 1000 replications; substitution model was set to p-distance for amino acid sequences; rates among sites were uniform, and patterns among lineages were homogeneous; gaps/missing data were treated with partial deletion (site coverage cutoff at 50%); and 9 threads were used for computational resource allocation. The generated phylogenetic tree was imported into ITOL (https://itol.embl.de/) and Adobe Illustrator for beautification, including branch adjustment, labeling of species names and annotation addition [36]. The Consurf web server was employed to analyze the evolutionary conservation of amino acid residues in QatD [37].

### Phylogenetic analysis of TatD-like protein and protein sequence alignment

The amino acid sequence of QatD was retrieved from the Pfam database to obtain the corresponding Pfam accession number (pfam01026) annotated for its functional domain [38]. Proteins with reviewed annotations were filtered from this entry, yielding a total of 66 sequences, which were downloaded in batch in FASTA format. The subsequent methods for constructing and beautifying the phylogenetic tree were consistent with those used in the phylogenetic tree analysis of the QatD protein. Sequence alignment was performed using ClustralW (https://www.genome.jp/tools-bin/clustalw) and illustrated with ESPript 3 [39].

### Phage cultivation and storage

Phages were propagated by picking a single phage plaque into a liquid culture of *E. coli* MG1655 grown at 37°C to an OD_600_ of 0.3–0.4 in LB medium supplemented with 10 mM MgCl_2_ and 2 mM CaCl_2_ until culture lysed. The culture was centrifuged for 20 min at 14 940 × *g* and the supernatant was filtered through a 0.2 μm filter to remove bacterial debris. The phages were then amplified and purified. The phage lysates were stored at 4°C for short-term use.

### Phage plaque assay

The reconstructed pET22b plasmid was transformed into BL21 (DE3) strain, a single colony was picked from the LB agar plate and grown in LB medium containing ampicillin (50 μg/ml) at 37°C to an OD_600_ of 0.6–0.8. Protein was induced with 0.2 mM IPTG, after overnight expression, 200 μl of the bacterial culture at OD_600_ 1.0 was mixed with 10 ml 0.5% top agar, and the samples were poured onto LB plates containing antibiotic. Plates were spotted with 5 μl of different phages diluted in MS buffer (100 mM NaCl, 10 mM MgCl_2_, 50 mM Tris–HCl) at 10-fold serial dilutions. Plates infected with λ and P1 phages were incubated at 37°C overnight, whereas plates for T1, T4, and T7 phages were incubated at 37°C for 6  h before imaging.

### Pull-down procedure

A total of 0.1 μM of each bait protein (GST-QatB, GST-QatC, with GST tag protein as the negative control) and 0.1 μM of prey protein (SUMO-QatD) were separately taken, diluted and mixed with binding buffer (20 mM HEPES, pH 8.0, 150 mM NaCl), and the total volume of the reaction system was adjusted to 400 μl. Then, 50 μl of pre-equilibrated GST affinity resin (GE Healthcare) was added to the mixture, followed by incubation at 4°C with gentle shaking for 4 h to promote the specific binding between proteins and the resin. After incubation, the resin pellet was collected by centrifugation at 800 × *g* for 30 s, and the supernatant was discarded. Subsequently, the resin was washed repeatedly 10 times with 0.5 ml of pre-cooled washing buffer [20 mM HEPES, pH 8.0, 150 mM NaCl, 0.1% (v/v) Triton X-100] to fully remove non-specifically bound contaminating proteins. The results were detected by SDS–PAGE electrophoresis.

## Results

### The Qat anti-phage system is widespread and exhibits anti-phage activity

**Figure 1.**
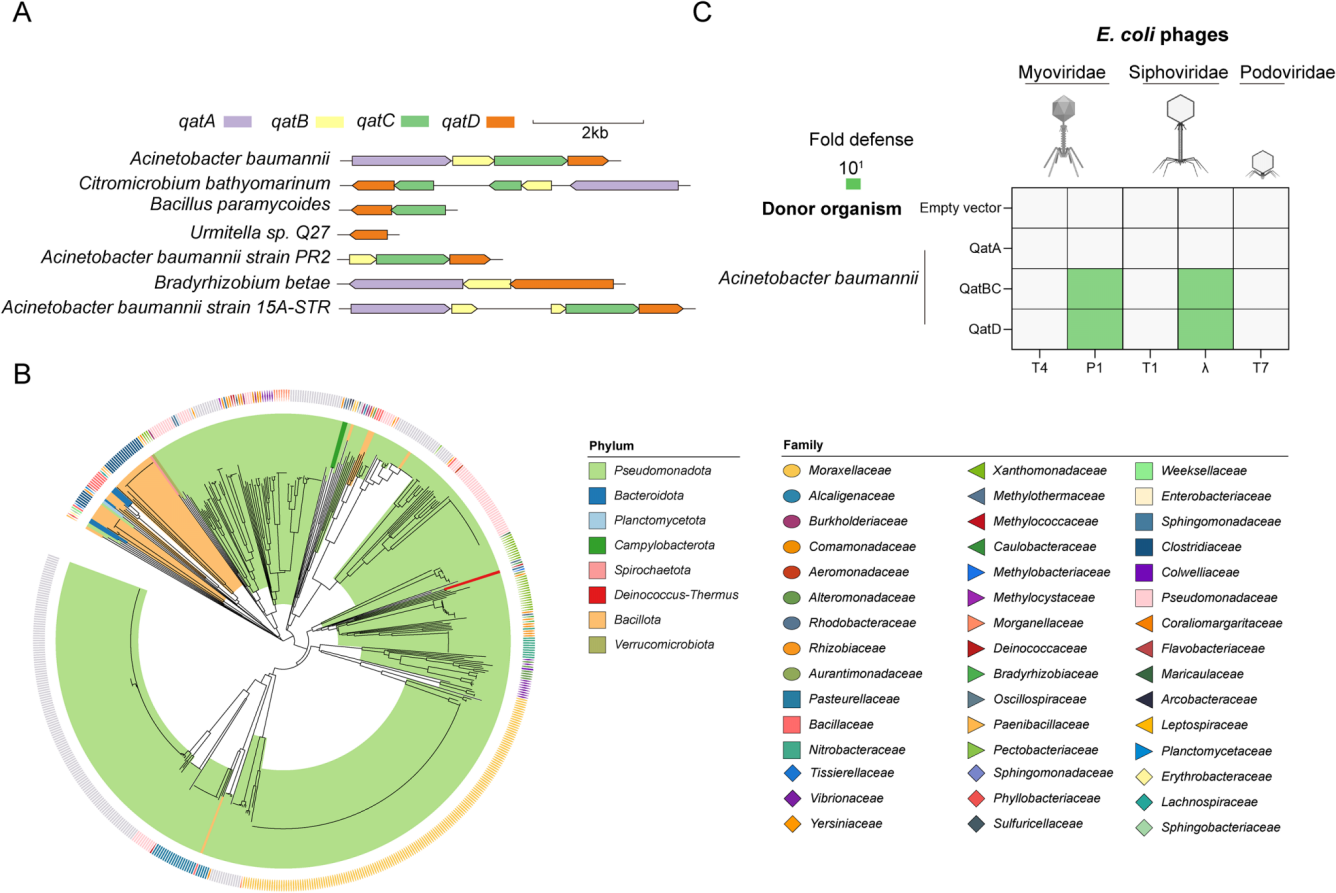
Qat anti-phage system is widespread. (**A**) The distribution and arrangement characteristics of the *qat* gene cluster and the scale bar (2 kb) denotes the length range of the gene cluster. Visualization was performed using ChiPlot (https://www.chiplot.online/). (**B**) The phylogenetic analysis results of QatD homologs across representative bacterial species. This circular phylogenetic tree uses sector colors to distinguish bacterial Phylum, while the color bars at the branch termini correspond to the more refined family (different colors and symbols in the legend denote phylum and family). (**C**) Heatmap showing fold defense of *E. coli* expressing Qat components (empty vector, QatA, QatBC, QatD) against phages λ, P1, T1, T4, and T7. Green indicates a 10-fold increase in resistance, all assays were performed in triplicate.

To investigate the distributional features and evolutionary dynamics of the Qat anti-phage defense system, we performed a systematic analysis of *qat* gene clusters across diverse bacterial lineages, including sequence comparison, structural annotation, and genomic context profiling. Our results reveal that the core *qatABCD* defense module exhibits a markedly modular and variable distribution pattern across species. We identified 7 distinct genomic existence forms, which are different combinations: for instance, *Bacillus paramycoides* retains only the *qatC* and *qatD* genes, while *Uncultured sp. Q27* carries solely a single *qatD* gene, resulting in highly simplified and incomplete operon configurations (Fig. 1A).

Further statistics show that 98.10% (*n* = 619) of the clusters correspond to the canonical full-length *qatABCD* operon, representing the overwhelmingly dominant form of this defense system in nature (Supplementary Fig. S1). In contrast, only a small fraction of loci exhibit truncated configurations: the *qatD*-only form accounts for 0.95% (*n* = 6), while the *qatBCD* submodule accounts for 0.32% (*n* = 2). Rare chimeric or incomplete variants, including *qatABBCD, qatCD, qatABD*, and *qatABCCD*, each represent <0.2% of all clusters (*n* = 1). The modular fragmentation and gene reduction patterns are likely shaped by repeated horizontal gene transfer (HGT) events and subsequent recombination during its dissemination among bacterial populations, enabling different hosts to retain distinct functional submodules according to their ecological pressures and antiviral requirements [40, 41].

To further explore how this modular organization relates to its evolutionary dissemination, particularly focusing on the frequently retained core components, we next analyzed the phylogenetic distribution of 631 QatD sequences across bacterial taxa (Fig. 1B). The phylogenetic tree reveals a highly uneven and lineage-biased distribution pattern of QatD. *Pseudomonadota* overwhelmingly dominates (91.7%), forming multiple densely packed clusters that display short branch lengths, indicating strong sequence conservation and potentially recent expansion within this phylum. In contrast, *Bacillota* (6.8%) forms several deeply branching clades that are clearly separated from the main *Pseudomonadota* clusters, suggesting that QatD in this phylum may have undergone ancient divergence or functional adaptation. The remaining phyla—including *Bacteroidota, Campylobacterota*, and *Spirochaetota*—are represented only by sporadic sequences that typically appear as isolated branches, indicating that these lineages are more likely derived from HGT rather than long-term vertical inheritance.

The natural existence of these fragmented *qat* modules (such as the *qatD*-only and *qatABCD* variants) and their diverse evolutionary trajectories prompted us to investigate whether these submodules possess independent defensive capabilities. Based on our preliminary analysis of the structural gene cluster, we evaluated the functions of the individual genetic components within the *qat* system using plaque assays (Fig. 1C). Challenge tests against a panel of five distinct bacteriophages (λ, P1, T1, T4, and T7) revealed that QatBC and QatD effectively conferred resistance against λ and P1 infections, whereas the other tested components exhibited no significant anti-phage activity (Supplementary Fig. S2). This experimental functional validation aligns with our genomic observations, highlighting QatD and QatBC as key autonomous functional units within the defense system.

### Overall structure of QatD

**Figure 2.**
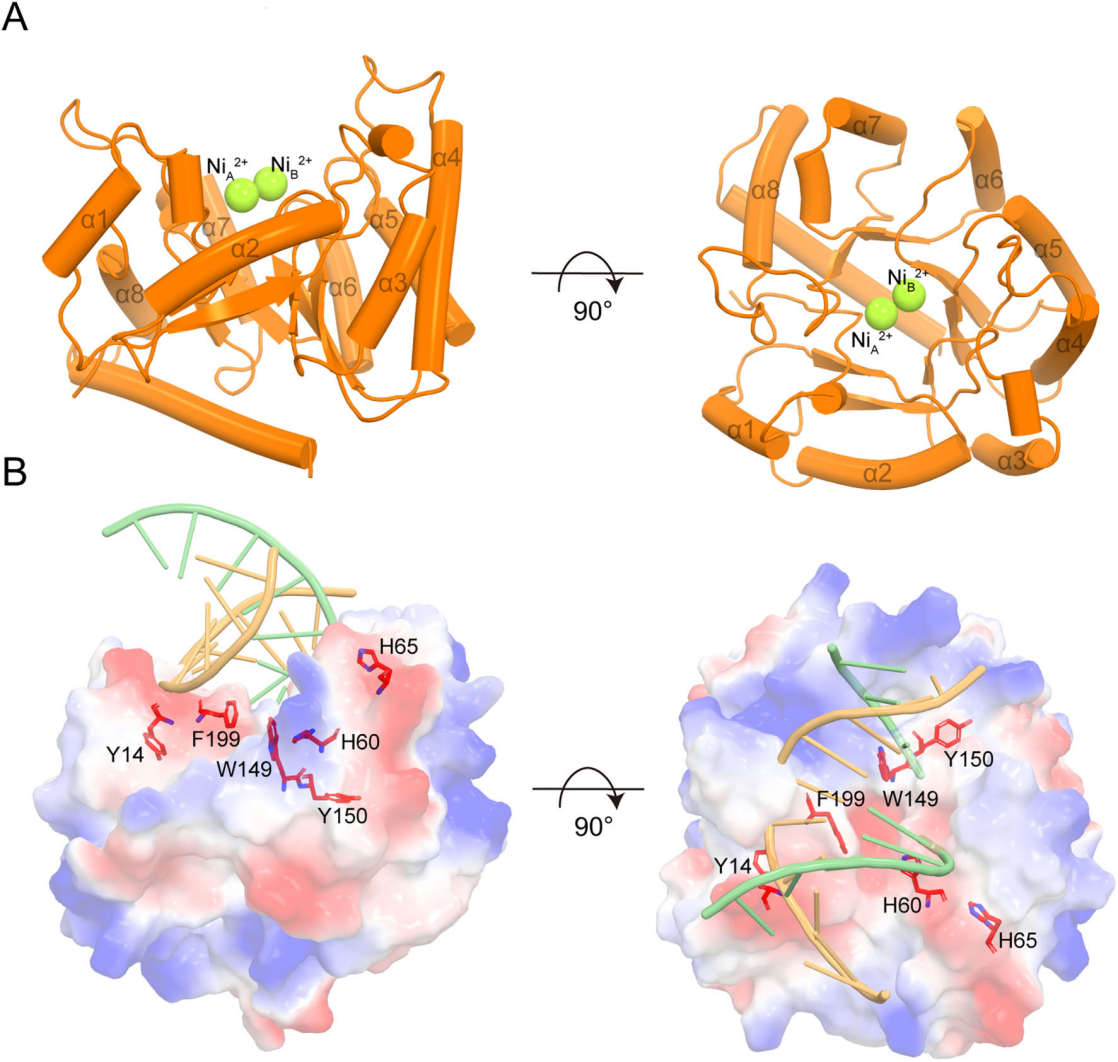
Structure characterization of QatD. (**A**) Crystal structure of QatD monomer, the limon spheres represent two Ni^2+^ ions bound in the active site, labeled Ni_A_^2+^ and Ni_B_^2+^. (**B**) Surface electrostatic potential map of the predicted QatD–DNA complex structure. The potential is colored from red (negative, −67.7 *kT/e*) to blue (positive, +67.7 *kT/e*), where brown/green structures represent the bound DNA molecule; the right subpanel displays the left complex rotated 90° downward along the *X*-axis. Spatial localization of critical amino acid residues involved in DNA recognition at the binding interface of QatD nuclease. Portions of the bound DNA colored green may appear discontinuous; this is purely a visual artifact resulting from the DNA backbone being partially obscured by the opaque representation of the protein’s surface.

The crystal structure of QatD (2.42 Å resolution) reveals a classic (β/α)_8_ TIM barrel architecture typical of TatD-family nucleases (PDB ID: 9XSN). Each monomer (chains A and B) is ~246 residues and folds into eight parallel β-strands forming an inner barrel, surrounded by nine α-helices on the outside (Fig. 2A). The two monomers in the asymmetric unit pack as a dimer, although gel-filtration data for QatD indicate the enzyme is monomeric in solution (Supplementary Fig. S3). The QatD overall structure closely resembles other TatD-family nucleases. Structural homology (DALI) shows QatD’s fold superimposes with high fidelity on *E. coli* TatD (RMSD ~0.7 Å over ~256 Cα) and on human and yeast TatD homologs (RMSD ~1.3–1.5 Å). Like these homologs, QatD’s TIM barrel features an α/β core typical of metal-dependent hydrolases. The barrel top—formed by the C-terminal ends of the β-strands—creates a distinct acidic pocket that hosts the catalytic center. This pocket contains the conserved metal-binding and catalytic residues typical of metal-dependent hydrolases (Fig. 2B). Within this pocket, strong electron density corresponding to two bound metal ions were observed. Although anomalous scattering data were not collected—precluding unambiguous identification of the metal species—systematic refinement testing of candidate divalent ions (such as Mg^2+^, Zn^2+^, and Ni^2+^) indicated that Ni^2+^ provided the best fit to the observed electron density. Given that QatD was purified by Ni-NTA affinity chromatography, the modeled Ni^2+^ most likely represents a purification-derived artifact rather than the native physiological cofactor.

Surrounding it, a broad basic surface enriched in conserved Lys/Arg residues occupies positions consistent with DNA-contact sites of other TatD nucleases, placing these side chains ideally for electrostatic engagement with the DNA phosphate backbone. Histidine residues such as H60 and H65 may further assist in orientating the DNA through hydrogen bonding to the backbone, while nearby aromatic residues could provide additional stabilization via base-stacking interactions (Fig. 2B). Together, these features define QatD as a canonical TatD-family TIM-barrel nuclease, in which a monomeric α/β core and loop extensions at the barrel top form a complementary electrostatic landscape that supports both DNA binding and catalysis.

### QatD is a 3′-5′ exonuclease

**Figure 3.**
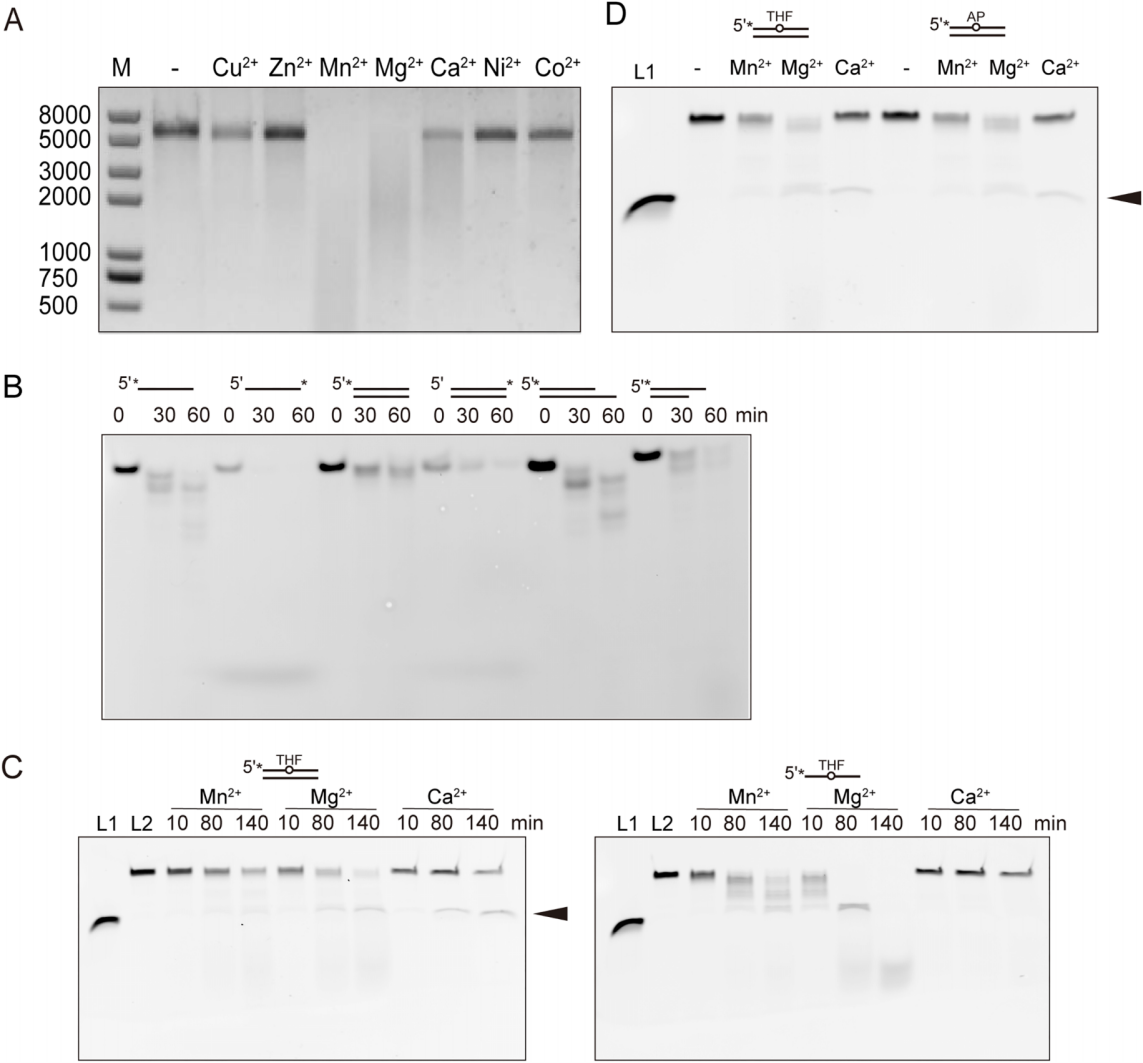
Nuclease activity of QatD. (**A**) Nuclease activity of QatD in the presence of different metal ions, including 10 mM CuCl_2_, 0.5 mM ZnCl_2_, 3 mM MnCl_2_, 10 mM MgCl_2_, 5 mM CaCl_2_, 1 mM NiCl_2_, 5 mM CoCl_2_ or 10 mM EDTA, 3.5 μM QatD were incubated 1 h at 37°C with 28.6 nM linear dsDNA fragment (PCR-amplified pET22b, 5360 bp). (**B**) Nuclease activity of 0.5 μM QatD on 0.1 μM various DNA substrates in the presence of 10 mM Mg^2+^, DNA substrates are labeled with 5′-FAM or 3′-FAM. Substrates heated to 70°C for 10 min were used as a control. Reaction products were analyzed by denaturing PAGE and visualized by fluorescence scanning. (**C**) Cleavage reactions were performed using double–stranded (left) and single–stranded (right) DNA substrates bearing a tetrahydrofuran (THF) abasic–site analog, in which 0.5 μM QatD was incubated with 0.1 μM substrate to achieve a 5:1 protein–to–DNA molar ratio. Lane 1 (L1) serves as a 12 bp size marker, while Lane 2 (L2) represents the negative control containing only the 25 bp DNA substrate without the addition of QatD protein. The 12 bp product band is indicated by a black triangle, all assays were performed in triplicate. (**D**) DNA containing natural AP sites is used for enzymatic cleavage experiments. 0.5 μM QatD were incubated 80 min at 37°C with 0.1 μM DNA. The 12 bp product band is indicated by a black triangle, all assays were performed with three independent replicates.

We tested the metal-ion dependence of QatD’s nuclease activity using a 5360 bp linear DNA fragment (PCR-amplified pET22b). In 1 h digests (3.5 μM QatD, 37°C), robust degradation occurred only in the presence of Mg^2+^ or Mn^2+^. Under MgCl_2_ or MnCl_2_, the intact 5360 bp band was completely lost and replaced by a smear of small fragments (Fig. 3A). In contrast, Ca^2+^ supported only weak activity—most DNA remained intact with only a slight smear—and Cu^2+^, Zn^2+^, Ni^2+^, or Co^2+^ supported virtually no cleavage (the full-length band remained unchanged). Therefore, QatD requires Mg^2+^ or Mn^2+^ for efficient nuclease function, with only partial activity in Ca^2+^. These results demonstrate that QatD is a metal-dependent nuclease with a clear preference for Mn^2+^ and Mg^2+^, consistent with the catalytic characteristics commonly observed among TatD family members [24, 42].

To determine the directionality and substrate preference of QatD, we next performed cleavage assays using single-stranded (ss) and dsDNA substrates labeled with either 5′- or 3′-FAM (Fig. 3B). Digestion of a 5′-FAM-labeled 25 mer yielded a ladder of progressively shorter fragments over time, whereas digestion of the same sequence labeled at the 3′ end yielded only a single short product band (a 3′-terminal nucleotide) and no internal fragments. These results indicate that QatD chewed from the 3′ end, releasing a discrete 3′-labeled mononucleotide while continually shortening the 5′-labeled substrate—exactly the pattern expected for a 3′-5′ exonuclease (Supplementary Fig. S4). Comparison of different substrate types further showed that QatD cleaved ssDNA more efficiently than dsDNA, suggesting a preferential activity toward ssDNA. Notably, QatD exhibited structure-specific rather than sequence-specific recognition. Its exonuclease activity was consistent across different oligonucleotide sequences with varying secondary structures, with no preferential cleavage observed at specific nucleotide motifs (Supplementary Fig. S5).

### QatD exhibit AP endonuclease activity

In addition to exonuclease activity, we also found that QatD incises abasic sites in DNA (AP endonuclease activity) with a strict metal-ion dependence. Using a 25 bp duplex containing a single synthetic abasic site analog (THF), we observed a clear binary switch in enzymatic activity, with 5 mM CaCl_2_, the THF-containing duplex was efficiently incised to yield a stable 5′fragment band. In contrast, in the presence of Mg^2+^ or Mn^2+^, the substrate band was gradually reduced over time. The minor bands observed near the 12 nt position in these lanes represent the accumulation of stalled intermediates from 3′-5′ exonucleolytic degradation at the AP lesion, rather than authentic endonucleolytic incision (Fig. 3C). This distinction was further confirmed using excess QatD at a 30:1 protein–to–DNA molar ratio. In the presence of Mg²⁺, exonucleolytic degradation stalls transiently at the THF–modified AP site but proceeds to completion over time. In contrast, Ca^2+^ specifically drives stable AP–site incision by QatD, generating a persistent cleavage product band with no further exonuclease–mediated degradation (Supplementary Fig. S6). This Ca^2+^-dependent AP endonuclease activity is highly specific for duplex DNA, as an analogous single-stranded THF-containing oligonucleotide remained intact under identical conditions.

Thus, QatD’s AP endonuclease activity is Ca^2+^-dependent and is specific for duplex DNA, not ssDNA—again mirroring TatD-family behavior (human TATDN1/3 show AP endonuclease only on dsDNA in Ca^2+^, and neither TATDN1 nor TATDN3 incise ssDNA AP sites) [25]. We also verified that QatD cleaves a naturally generated abasic (AP) site. A 25 mer duplex containing a single uracil was first treated with UDG to generate an authentic AP lesion, and the resulting substrate was then incubated with QatD (0.5 μM). Consistent with the synthetic AP substrate, the specific AP endonuclease activity is robustly activated by Ca^2+^ (Fig. 3D). Under these conditions, the full-length band was largely abolished, indicating efficient incision at the AP site. In contrast, the AP lesion remained predominantly intact in Mg^2+^ or Mn^2+^ buffers. This Ca²⁺-dependent processing of a bona fide AP site demonstrates that QatD possesses authentic AP endonuclease activity.

Overall, QatD joins the TatD/TATDN family as a dual nuclease. Like *E. coli* TatD, QatD is a 3′-5′ exonuclease with strict Mg^2+^/Mn^2+^ dependence and shows no requirement for high concentrations of Zn^2+^ or other transition metals [24]. However, unlike TatD, QatD retains at least weak activity in Ca^2+^—in fact Ca^2+^ switches QatD into an AP-cleavage mode, as noted for human TATDN1/3. Human TATDN1 and TATDN3 also exhibit this duality, preferring to remove 3′-blocks on ssDNA but cleaving duplex abasic sites in Ca^2+^ [25]. In keeping with this, we observed that QatD, like TatD-family enzymes, degrades single- or double-stranded DNA in a 3′-5′ direction, whereas prototypical AP endonucleases (e.g. APE1 or bacterial Endonuclease IV) are optimized for double-stranded substrates and display little 3′-exonucleolytic trimming [43–45]. Thus, QatD’s cofactor requirements and substrate preferences closely parallel those of other TatD-like nucleases and distinguish its activity from classic AP endonucleases.

### QatD is a Type-II TatD nuclease that employs two-metal catalysis

**Figure 4.**
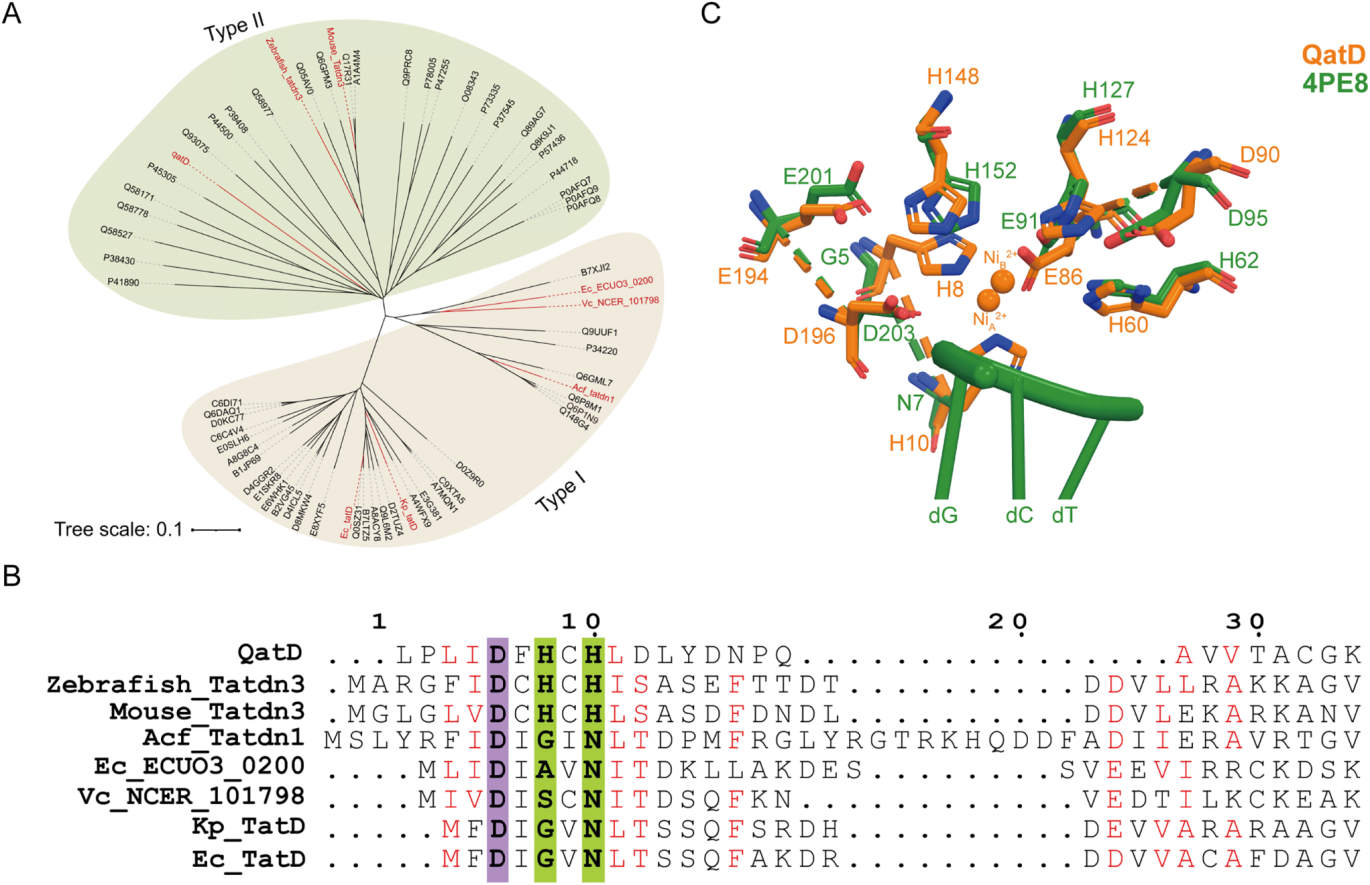
Active sites of QatD. (**A**) Phylogenetic tree of TatD related proteins, constructed based on amino acid sequence alignment, proteins in the tree are clustered into two major clades (Type I, Type II) according to their evolutionary relatedness; each node corresponds to a TatD-related protein from different species. The red branches represent representative protein subgroups selected from each of the two clades. (**B**) Partial sequence alignment of the N-terminal region of QatD orthologs from *A. baumannii, Zebrafish* (Zebrafish_Tatdn3), *Mouse* (Mouse_Tatdn3), *African clawed frog* (Acf_Tatdn1), *Encephalitozoon cuniculi* (Ec_ECUO3_0200), *Vairimorpha ceranae* (Vc_NCER_101 798), *Klebsiella pneumoniae* (Kp_TatD) and *E. coli* (Ec_TatD). The (G/S/A)xN (Type I) and HxH (Type II) are highlighted. (**C**) Superimposition of the QatD and *E. coli* TatD active sites. QatD (PDB ID: 9XSN) is colored orange, and *E. coli* TatD bound to a d (GCT) trinucleotide (PDB ID: 4PE8) is colored green. The conserved catalytic residues exhibit near-perfect spatial alignment. The overlaid d (GCT) substrate (green) from the 4PE8 complex docks adjacent to the two Ni^2+^ ions (orange spheres) of QatD.

Chen *et al*. classified TatD-family nucleases into two subgroups based on key active-site residues [24]. Type I members contain the (G/A/S)xN motif and typically harbor a single metal-binding site, whereas Type II proteins possess the characteristic HxH motif, which supports stable coordination of two metal ions. To assess where QatD falls within this framework, we constructed a phylogenetic tree using 66 TatD homologs. The resulting topology clearly separates all TatD proteins into two distinct clades (Fig. 4A), consistent with the previously reported subgroup division. QatD clusters within the Type II clade and contains the conserved HxH motif, indicating from sequence features (Fig. 4B) alone that it is a Type II enzyme and therefore capable of binding two metal ions.

Crystallographic analysis provides direct structural confirmation of this prediction. The QatD structure solved in the presence of Ni^2+^, reveals a fully occupied binuclear metal center in the active site. These occupancy patterns closely resemble those of other Type II TatD enzymes—such as *S. aureus* TatD (two Ni^2+^, PDB ID: 2GZX) and human TATDN3 (two Zn^2+^, PDB ID: 2Y1H) —which are known to employ a two-metal catalytic mechanism. In QatD, each metal ion is coordinated by one pair of HxH–motif histidine residues (either His–8/His–10 or His–124/His–148), together with a neighboring acidic amino–acid ligand. Notably, Glu-86 appears to bridge the two metal ions, with its two carboxylate oxygens contacting distinct Ni^2+^ positions. To further contextualize this bimetallic center, we superimposed the QatD active site with the *E. coli* TatD-nucleotide complex (PDB ID: 4PE8). This structural alignment demonstrates that the d (GCT) substrate docks perfectly adjacent to the binuclear center of QatD (Fig. 4C). The resulting coordination geometry matches that of established TatD-family and related metallophosphoesterase-like bimetallic nucleases, supporting a universally conserved mechanism in which a bridging water or hydroxide located between the two metals is activated to attack the scissile phosphodiester bond [46].

### Active-site architecture of QatD

**Figure 5.**
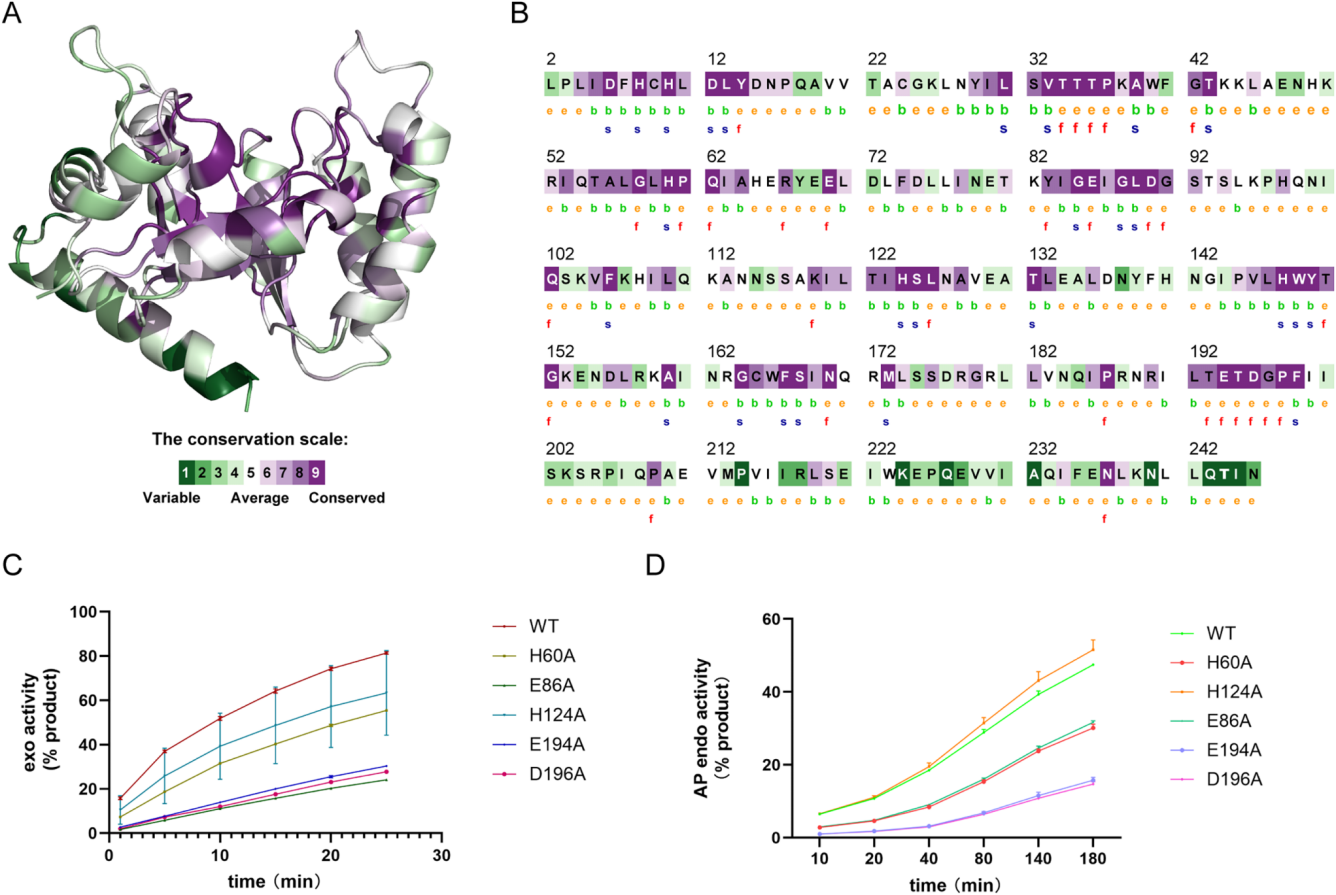
Evolutionary conservation analysis and functional validation of key residues in QatD. (**A**) Surface Representation of Protein Evolutionary Conservation. The conservation scores, calculated from multiple sequence alignment, are mapped onto the 3D structural model of the protein. The color scale ranges from variable (dark green, score 1) to highly conserved (dark purple, score 9). (**B**) Sequence-based conservation and secondary structure prediction. Detailed view of the primary amino acid sequence with associated conservation scores (highlighted in purple/green). Symbols below the sequence indicate predicted secondary structural elements and solvent accessibility [e, an exposed residue according to the neural network algorithm; b, a buried residue according to the neural network algorithm; f, a predicted functional residue (highly conserved and exposed); s, a predicted structural residue (highly conserved and buried)]. (**C**) Kinetic analysis of the exonuclease (exo) activity of QatD and its mutants. The percentage of substrate product formation was monitored over 30 min. The results show that the wild-type (WT) possesses the highest exonuclease activity, while mutations at different sites result in varying degrees of activity reduction. (**D**) Kinetic analysis of the AP endonuclease (AP endo) activity of QatD and its mutants. The cleavage efficiency of each protein toward AP-site-containing substrates was measured over 180 min. Data points represent the mean of three independent experiments, and error bars indicate the standard deviation (SD).

To further investigate the structural basis of QatD’s enzymatic function, we first performed an evolutionary conservation analysis using the ConSurf server. By mapping the conservation scores onto the crystal structure of QatD, we observed a striking correlation between sequence conservation and the functional architecture of the protein (Fig. 5A). The analysis revealed that the most highly conserved residues (dark purple, scores 8–9) are not randomly distributed but are predominantly clustered within the internal cavity and the top-loop region of the (β/α)_8_ TIM barrel (Fig. 5A). This conserved core coincides precisly with the predicted catalytic center, while the peripheral surfaces exhibit significantly higher sequence variability (dark green, scores 1–3) (Fig. 5B). This structural conservation pattern strongly suggests that the top-loop region forms a specialized active site optimized for metal coordination and phosphodiester hydrolysis.

Consistent with these structural insights, the active site of QatD is located in the barrel’s top-loop region and is composed of a constellation of conserved acidic and histidine residues. The N-terminal HxH motif of QatD (His-8 and His-10) directly coordinates Metal 1, effectively replacing the Gly and Asn found at equivalent positions in other Type-I TatD homologs. By analogy to *E. coli* TatD numbering, the binuclear active site is assembled from the following conserved ligands: Glu-86 serves as a bridging residue coordinating both Metal 1 and Metal 2 (corresponding to *Ec*TatD Glu-91); Asp-196 contributes an additional contact to Metal 1 (corresponding to *Ec*TatD Asp-203); and two interior histidines, His-124 and His-148 (analogous to *Ec*TatD His-127 and His-152), coordinate Metal 2 together with the bridging residue Glu86, forming an arrangement consistent with the canonical binuclear active-site geometry observed in the Type-II TatD-family homologs 1YIX and 2GZX. His-60 (equivalent to *Ec*TatD His-62) is positioned as a catalytic residue rather than a metal coordinator; mutation of this histidine in *E. coli* abolishes nuclease activity [24], indicating it likely acts as a general base to activate the attacking water molecule for phosphodiester hydrolysis (Supplementary Fig. S7).

To investigate the regulatory role of these amino acid residues in QatD enzymatic activity, we constructed multiple mutants, including QatD^H8A^, QatD^H10A^, QatD^H60A^, QatD^E86A^, QatD^D90A^, QatD^H124A^, QatD^H148A^, QatD^E194A^, and QatD^D196A^. Due to poor solubility, the QatD^H8A^, QatD^H10A^, QatD^D90A^, and QatD^H148A^ variants failed to yield soluble protein and were excluded from enzymatic assays. The complete loss of solubility upon mutating these conserved metal-coordinating residues strongly suggests that their side chains, and likely the structural incorporation of the divalent metal ions themselves, are absolutely essential for the proper folding and structural integrity of the QatD (β/α)_8_ TIM-barrel core. The remaining mutants were purified and subjected to nuclease activity measurements.

We assessed 3′-5′ exonuclease activity using a 3′-FAM-labeled ssDNA substrate in the presence of Mg^2+^, and AP endonuclease activity using a 5′-FAM-labeled dsTHF substrate in the presence of Ca^2+^ (Supplementary Fig. S8). Enzymatic activity assays demonstrated that substitutions at the catalytic core residues E194A and D196A resulted in a significant abrogation of nuclease activity, consistent with their critical spatial positions coordinating the metal ions in the QatD structure. In contrast, QatD^H60A^ and QatD^E86A^ mutants exhibited a moderate reduction in enzymatic activity, indicating their partial but non-essential roles in catalysis. Notably, the QatD^H124A^ mutant retained nearly wild-type levels of AP endonuclease activity while only showing a slight decrease in exonuclease activity (Fig. 5C and D). This differential impact on exonuclease and AP endonuclease activities strongly suggests that H124 plays a specialized role in the substrate recognition or catalytic steps specific to the exonuclease reaction, rather than being a universal catalytic residue.

### The exonuclease activity of QatD is modulated by intracellular NTPs

We previously demonstrated that QatD is a bifunctional nuclease with both 3′-5′ exonuclease and AP-endonuclease activities. However, the molecular mechanism underlying its anti-phage function remains unclear. Nucleases in many anti-phage defense systems are tightly regulated by signaling molecules to prevent aberrant activation and host toxicity. For example, NucC is activated by cyclic trinucleotides and assembles into a hexameric complex to cleave DNA, while the endonuclease activity of the Gabija immune system is inhibited by physiological concentrations of NTPs [47, 48].

**Figure 6.**
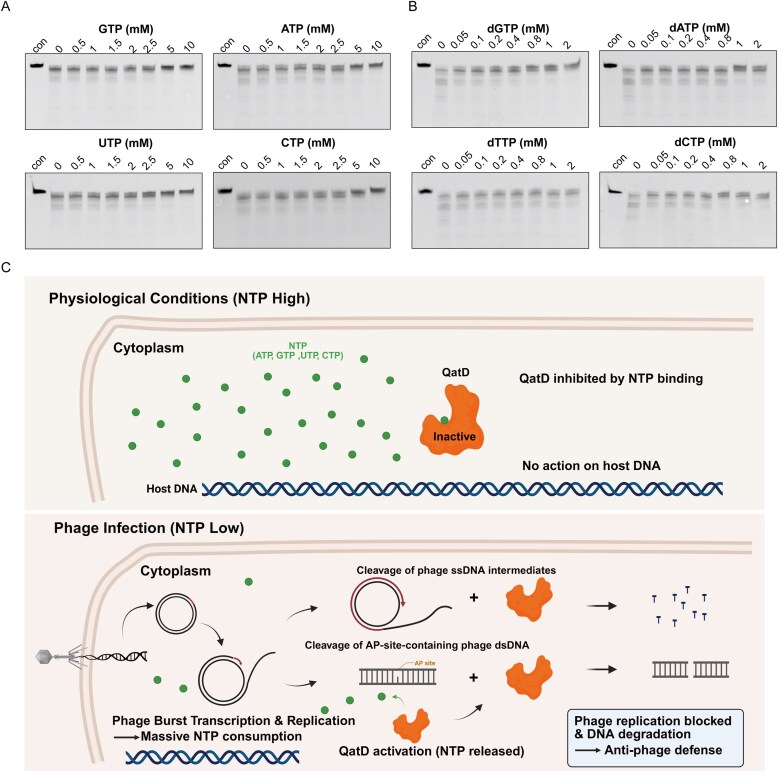
QatD exonuclease activity is inhibited by NTPs but not dNTPs. (**A**) The exonuclease activity of QatD was assayed using a 5′-FAM-labeled ssDNA substrate. Reactions containing 0.5 μM QatD and 0.1 μM substrate in the presence of 10 mM Mg^2+^ were incubated at 37°C for 20 min, supplemented with the indicated concentrations of each NTPs (0–10 mM). Each assay was conducted in triplicate. (**B**) The exonuclease activity of QatD was assayed using a 5′-FAM-labeled ssDNA substrate. Reactions containing 0.5 μM QatD and 0.1 μM substrate in the presence of 10 mM Mg^2+^ were incubated at 37°C for 30 min, supplemented with the indicated concentrations of each dNTPs (0–2 mM). All assays were performed in three independent replicates. (**C**) Model of NTP–dependent regulation of QatD anti–phage activity. High cellular NTP keep QatD inactive; phage–induced NTP depletion activates QatD to degrade phage DNA and inhibit phage propagation.

To investigate whether QatD is similarly regulated by cellular nucleotides, we tested the effect of varying concentrations of NTPs (ATP, GTP, CTP, UTP) and dNTPs (dATP, dGTP, dCTP, dTTP) on its nuclease activity *in vitro*. In the absence of nucleotides (0 mM), QatD efficiently degraded the ssDNA substrate, as evidenced by the reduction of the full-length band and the appearance of shorter degradation products. Addition of NTPs caused a clear dose-dependent inhibition of this activity: significant inhibition was observed at 2–5 mM, and activity was nearly abolished at 10 mM (Fig. 6A). These concentrations overlap with or are close to the physiological NTP levels in exponentially growing *E. coli* (ATP ~9.6 mM, UTP ~8.3 mM, CTP ~2.7 mM, GTP ~4.9 mM). In contrast, dNTPs, which are maintained at much lower physiological concentrations (dATP ~181 μM, dCTP ~184 μM, dGTP ~92 μM, dTTP ~256 μM), only weakly inhibited QatD activity within the tested range up to 2 mM [49, 50] (Fig. 6B).

This NTP-dependent inhibition is reminiscent of the regulatory mechanisms reported for other anti-phage nucleases, such as those in the Ppl, Septu, and KELShedu systems [51–53]. Based on these results, we propose a potential model for QatD activation during phage infection. Phage burst transcription and replication impose a heavy demand on cellular nucleotide pools (NTPs for massive RNA synthesis and energy, dNTPs for genome replication), which may lead to local depletion of intracellular NTPs. Such a reduction could relieve NTP-mediated inhibition of QatD, unleashing its nuclease activity to target invading phage DNA. Specifically, the ssDNA intermediates generated during phage replication (e.g. the single-stranded tails produced by P1 phage rolling-circle replication) would be susceptible to degradation by QatD’s 3′-5′ exonuclease activity. Additionally, QatD’s AP-endonuclease activity may selectively cleave AP sites formed in the dsDNA phage genome during rapid replication. This precise division of labor perfectly aligns with our *in vitro* finding that the AP endonuclease activity is strictly specific for duplex DNA, thereby contributing to the suppression of phage propagation (Fig. 6C).

## Discussion

In this study, we systematically characterized the structural and biochemical basis of QatD function, establishing it as a metal-dependent dual nuclease, Mg^2+^-dependent 3′-5′ exonuclease activity and Ca^2+^-dependent AP endonuclease activity, that has been evolutionarily repurposed for antiphage defense.

A central question for all nuclease-based immune systems is how self-DNA is protected from degradation while foreign DNA is selectively targeted. Our results shed light on this dilemma by uncovering a dual-layered self/non-self discrimination mechanism operating at both structural and metabolic levels: (i) structural gating, through its strict requirement for an accessible free 3′ terminus, which the intact circular host chromosome does not present under normal conditions; and (ii) metabolic licensing, through NTP-dependent inhibition, which couples enzymatic activation to the physiological state of the infected cell. Although inhibition requires NTP concentrations in the millimolar range, this threshold closely matches the physiological nucleotide pool in healthy cells, thereby minimizing the risk of autoimmunity and ensuring that QatD is activated only under the profound nucleotide depletion accompanying phage replication. Furthermore, while QatD exhibits robust Ca^2+^-dependent AP endonuclease activity *in vitro* at optimal millimolar concentrations, physiological intracellular free Ca^2+^ remains fundamentally lower (~90 nM) [54]. This striking physiological disparity suggests that localized Ca^2+^ perturbation during phage infection is unlikely to act as the primary *in vivo* trigger, underscoring the central role of this NTP-mediated metabolic gate.

This two-tiered mechanism allows QatD to mount a selective and timely antiphage response while preserving the integrity of the host genome during normal growth. Upon phage infection, rapid viral DNA transcription and DNA replication drastically increase nucleotide consumption, leading to localized depletion of cellular NTP pools. This metabolic crisis relieves NTP-mediated inhibition of QatD, unleashing its dual nuclease activities to target invading phage genomes. QatD’s 3′-5′ exonuclease preferentially degrades the abundant ssDNA intermediates generated during phage replication, such as the ssDNA tails produced by P1 rolling-circle replication. Concurrently, the high replication error rate and oxidative stress during viral propagation promote the accumulation of a high density of abasic (AP) sites within the double-stranded regions of the phage genome [55, 56]. Unlike the sparse abasic lesions on the host chromosome that are rapidly sequestered and processed by endogenous base excision repair machinery, these heavily damaged and unrepaired viral genomes serve as the primary, high-affinity substrate pool for unleashed QatD. QatD’s Ca^2+^-dependent AP endonuclease activity selectively cleaves these damaged sites, disrupting phage DNA integrity and blocking productive infection. In this manner, QatD couples metabolic sensing with dual DNA-cleaving activities to mount a potent and selective antiviral response (Fig. 6C).

The physical interaction between QatD and QatB, although weak, was detected by our GST pull-down assay, provides an important structural link between QatD and the core QatBC complex, and raises intriguing questions about the functional coordination of these components during phage infection (Supplementary Fig. S9). Notably, recent landmark work by Gao *et al*. and Oh *et al*. have demonstrated that the QatBC complex alone is sufficient to independently confer baseline protection against specific dsDNA phages, establishing QatBC as a functionally autonomous defense module [14, 15]. Our biochemical data further demonstrate that QatD possesses intrinsic nuclease activity, including its characteristic NTP-dependent inhibition, that does not require association with other Qat components *in vitro*. The QatABCD system therefore appears to employ a modular defense architecture in which QatBC and QatD represent distinct yet physically interacting effector units.

Within this framework, we propose that the QatD–QatB interaction may serve to spatially coordinate the two defense layers at the site of phage attack, rather than to activate either component per se. When phage infection triggers NTP depletion, QatD is relieved from inhibition and its nuclease activity is unleashed; physical tethering to QatB could concentrate QatD in proximity to the QatBC defense complex, thereby enhancing the efficiency and breadth of the composite anti-phage response. In this model, QatBC and QatD act as parallel but coordinated effectors. QatBC providing baseline protection while QatD contributes an orthogonal nuclease function targeting AP sites or linear DNA intermediates generated during phage replication. With their physical association ensuring a spatially integrated rather than independent and uncoordinated response. This cooperative arrangement may also explain why QatD provides additional anti-phage activity beyond that conferred by QatBC alone, as observed in our plaque assays. However, our challenge assays also revealed that the Qat system does not confer broad-spectrum resistance against all tested phages, specifically failing to protect against T1, T4, and T7 (Fig. 1C). This highlights the functional boundaries of this defense mechanism. For instance, the T4 genome is heavily modified with hydroxymethyl cytosine and subsequent glycosylation, which might introduce severe steric hindrance that blocks QatD from accessing the DNA backbone or AP sites [27, 57]. Alternatively, phages like T7, which possess extremely rapid replication cycles and encode their own highly efficient machineries, might either outpace the NTP depletion threshold required for QatD activation or encode specific, yet-unidentified anti-defense proteins that antagonize the Qat system [58].

At the molecular level, QatC has recently been shown to catalyze ATP-dependent deazaguanylation of QatB itself [14, 15]. Building on this finding together with our own observations, it is tempting to speculate that this QatC-mediated modification could influence the affinity or geometry of the QatD-QatB interaction, providing a mechanism by which the upstream enzymatic activity of QatC fine-tunes the recruitment or positioning of QatD within the defense complex. Such a regulatory relay would elegantly couple the activation state of the full QatABCD system to a coordinated, multi-layered nuclease response against invading phage DNA. This model remains speculative and awaits experimental validation; nevertheless, it highlights the QatB–QatD physical interface as a priority target for future structural and functional investigation.

## Supplementary Material

gkag776_Supplemental_File

## Data Availability

Coordinates of the X-ray crystal structures have been deposited in the RCSB PDB (www.rcsb.org) with the following accession numbers: QatD (https://doi.org/10.2210/pdb9xsn/pdb). Other data are available upon reasonable request.
