# Supplementary material for: Structural and enzymatic insights into QatD, a dual-function TatD-like nuclease in the QatABCD anti-phage defense system
Source: Nucleic Acids Res. 2026 Aug 4;54(14):gkag776. doi: 10.1093/nar/gkag776 (PMC13434339; doi:10.1093/nar/gkag776)
Supplement: Supplementary file 1 [file gkag776_supplemental_file.pdf]

Supplementary data for

Structural and Enzymatic Insights into QatD, a dual-function TatD-like Nuclease in the QatABCD Anti-Phage Defense System

Xu Wang[a†], Na Wang[a†#], Lin Zhang[b†], Meilin Zhang[a], Yaling Xu[a], Zheng Cao[b], Honghua Ge[a#], Jinming Ma[a#]

[a] School of Life Science and Medical Engineering, Anhui University, Hefei 230601, China

[b] Beijing Obstetrics and Gynecology Hospital, Capital Medical University. Beijing Maternal and Child Health Care Hospital. No.251 Yaojiayuan Rd, Beijing 100026, China

# Correspondence email: wangn6@ahu.edu.cn, hhge@ahu.edu.cn, jmma@ahu.edu.cn

† These authors contributed equally to this work.

Supplementary Table 1. Plasmids used in this study.

**Plasmid Name**	**Use**	**Source**
pET22b	Negative control for Phage plaque assay	Lab stock
pGEX_GST	Protein expression	Current study
pGEX_GST-QatB	Protein expression	Current study
pGEX_GST-QatC	Protein expression	Current study
pET22b_SUMO-QatD	Protein expression	Current study
pET22b_QatA	Phage plaque assay	Current study
pET22b_QatBC	Phage plaque assay	Current study
pET22b_QatD	Protein expression and Phage plaque assay	Current study
pET22b_QatD^{H8A}	Protein expression	Current study
pET22b_QatD^{H10A}	Protein expression	Current study
pET22b_QatD^{H60A}	Protein expression	Current study
pET22b_QatD^{E86A}	Protein expression	Current study
pET22b_QatD^{D90A}	Protein expression	Current study
pET22b_QatD^{H124A}	Protein expression	Current study
pET22b_QatD^{H148A}	Protein expression	Current study
pET22b_QatD^{E194A}	Protein expression	Current study
pET22b_QatD^{D196A}	Protein expression	Current study

Supplementary Table 2. X-ray data collection and refinement statistics.

**Data collection statistics**	
Wavelength	0.9793 Å
Resolution range	36.37 - 2.42 (2.51 - 2.42)
Space group	$P2_12_12_1$
a, b, c (Å)	57.15 64.99 141.45
α, β, γ (Å)	90.00 90.00 90.00
R_{meas}	0.16 (1.33)
Mean I/sigma(I)	17.84 (2.02)
$CC_{1/2}$	0.99 (0.78)
Completeness (%)	99.70 (98.83)
Multiplicity	12.30 (11.69)
Wilson *B*-factor (Å)	63.21
**Refinement statistics**	
Resolution range	36.37 - 2.42 (2.51 - 2.42)
Unique reflections	20705 (2027)
R_{work}	0.25 (0.39)
R_{free}	0.27 (0.43)
Number of non-hydrogen atoms	3733
Macromolecules	3708
Ligands	11
Solvent	14
Average *B*-factors (Å)	85.13
Macromolecules	85.22
Ligands	86.37
Solvent	58.95
RMSD bonds (Å)	0.003
RMSD angles (deg)	0.60

[a] Statistics for the highest-resolution shell are shown in parentheses.

Supplementary Table 3. Sequences of oligonucleotides used.

Model	Sequence (5′⟶3′)
5’ * 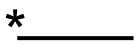	FAM-CGTCTCCTGAACTGTGCTATCGCTC
5’ *	CGTCTCCTGAACTGTGCTATCGCTC-FAM
5’ * 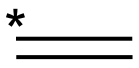	FAM-CGTCTCCTGAACTGTGCTATCGCTC<br>GCAGAGGACTTGACACGATAGCGAG
5’ *	CGTCTCCTGAACTGTGCTATCGCTC-FAM<br>GCAGAGGACTTGACACGATAGCGAG
5’ * 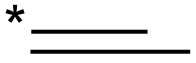	FAM-CGTCTCCTGAACTGTGCTATCGCTC<br>GCAGAGGACTTGACACGATAGCGAGAAAAAAAA
5’ * 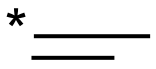	FAM-CGTCTCCTGAACTGTGCTATCGCTC<br>GCAGAGGACTTGACACG
5’ * 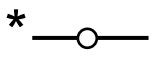	FAM-CGTCTCCTGAAC/(THF)/GTGCTATCGCTC
5’ * 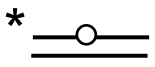	FAM-CGTCTCCTGAAC/(THF)/GTGCTATCGCTC<br>GCAGAGGACTTGACACGATAGCGAG
5’ *	FAM-CGTCTCCTGAAC

Name	Sequence (5′⟶3′)
D1	FAM-CTATAGAGGGACAAACTCAAGGTCATTCGCA<br>AGAGTGGCCTTTATGATTGACCTTCTC
D2	FAM-CGACTCACTATAGAGGGACAAACTCAAGGTC<br>ATTCGCAAGAGTGGCCTTTATGATTGACCTTC<br>TTCCGGTTAATACGAC
D3	FAM-AAACTCAAGGTCATTCGCAAGAGTGGCCTTTA<br>TGATT

Table Note:

*: 6-carboxyfluorescein (FAM) fluorophore.

○: Tetrahydrofuran abasic site analog.

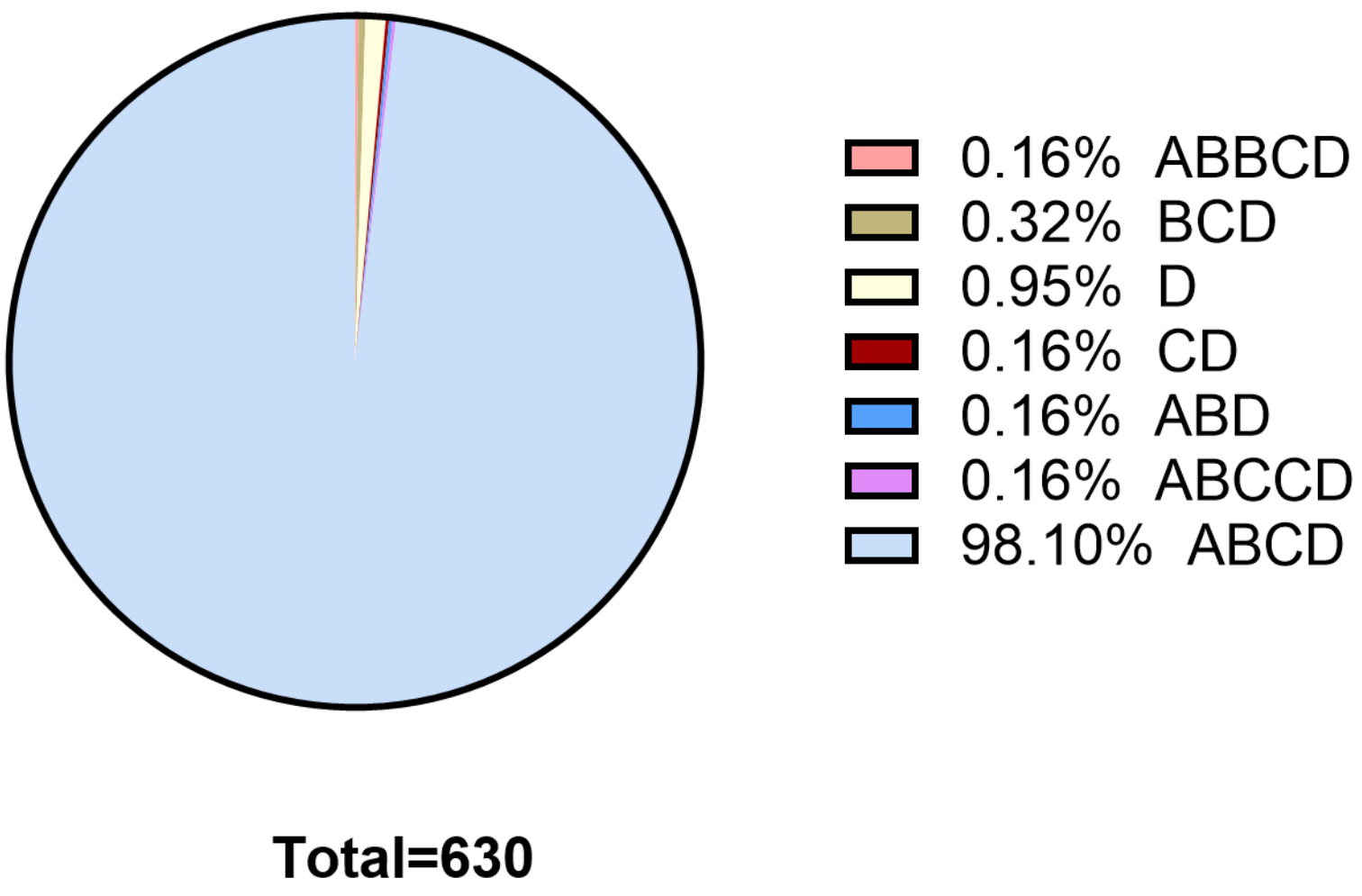

Supplementary Figure 1. Distribution of qat gene cluster configurations among 631 identified loci. Pie chart showing the proportion of different *qat* operon configurations. The canonical full-length *qatABCD* operon accounts for 98.10% (n=619) of all loci, while truncated and chimeric variants are rare (each ≤0.95%).

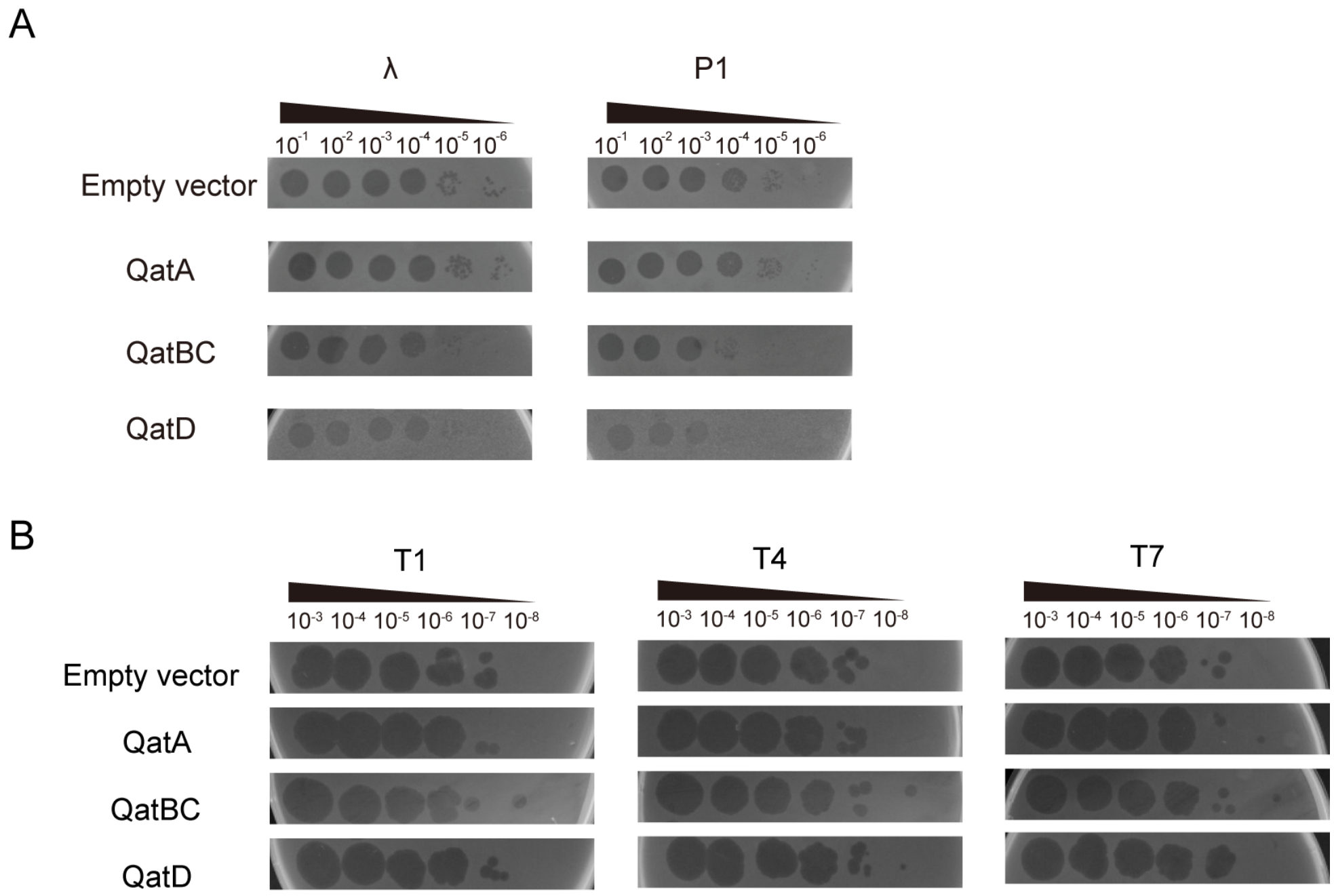

Supplementary Figure 2. Representative plaque-forming assays showing the anti-phage activity of individual Qat components against diverse phage strains. **A)** Plaque assays for λ and P1 infecting cells expressing empty vector, QatA, QatBC, or QatD. Serial 10-fold phage dilutions are indicated above each blot. **B)** Plaque assays for T1, T4, and T7 against the four indicated constructs, with serial 10-fold phage dilutions as labeled.

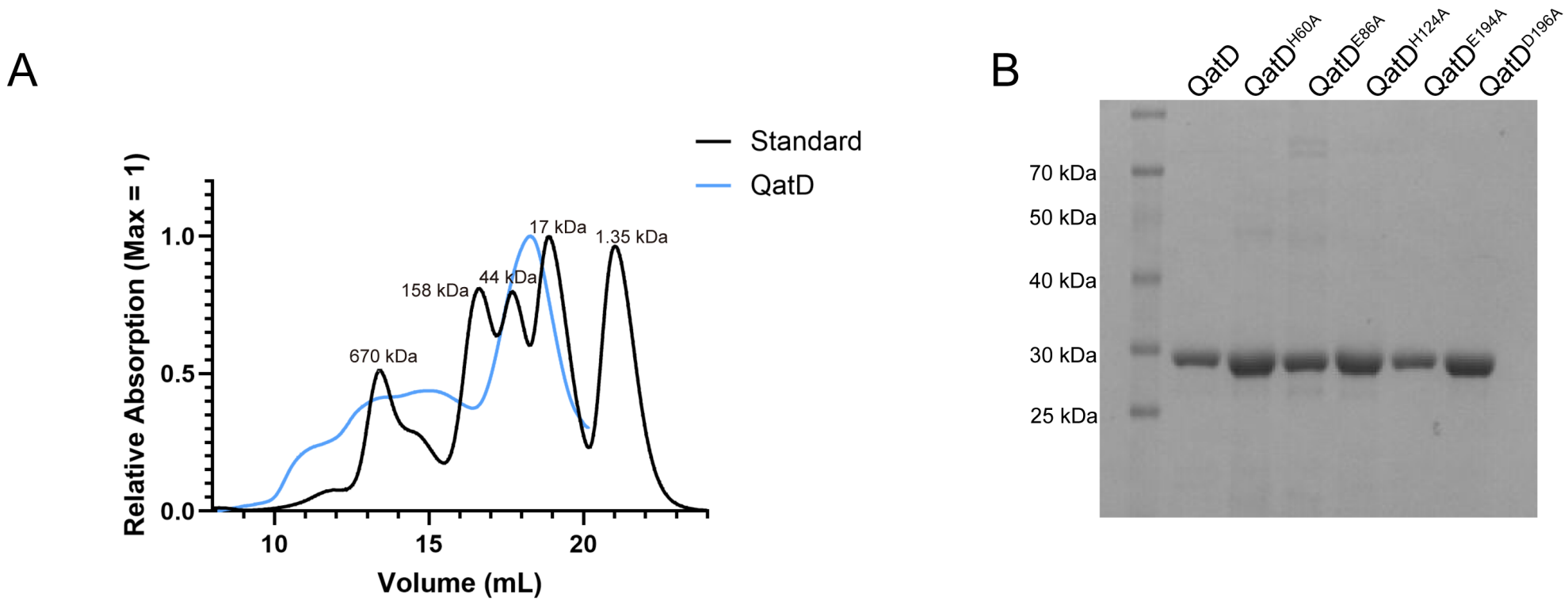

Supplementary Figure 3. Biochemical characterization of QatD and its mutants. **A)** Size-exclusion chromatography (SEC) analysis of QatD, where the blue curve represents the purified QatD protein and the black curve represents the standard reference proteins of known molecular weights (670 kDa, 158 kDa, 44 kDa, 17 kDa, 1.35 kDa); the elution peak of QatD indicates that it exists as a monomer in solution. **B)** SDS-PAGE analysis of QatD and its point mutants (1.0 mg/mL for H60A, H124A, and D196A; 0.6 mg/mL for wild-type and remaining variants), with the protein molecular weight marker shown on the left; the results demonstrate that both the wild-type and all mutants were purified to homogeneity, with an apparent molecular weight of approximately 30 kDa, consistent with the predicted monomeric size.

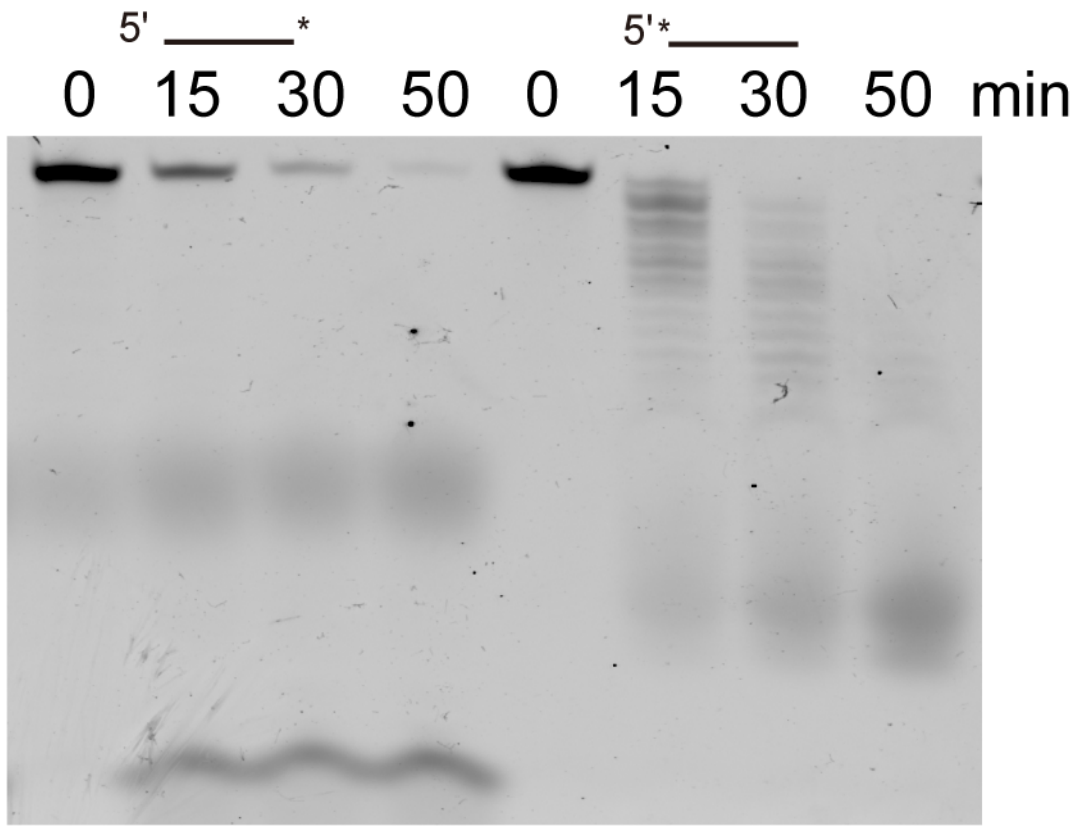

Supplementary Figure 4. Nuclease activity of QatD against single-stranded DNA (ssDNA). Urea-denaturing polyacrylamide gel electrophoresis analysis of ssDNA degradation by QatD protein over time. The ssDNA substrates, labeled with FAM at either the 3'-end (left lanes) or the 5'-end (right lanes), were incubated with purified QatD in the presence of 10 mM Mg^{2+} for the indicated durations (0, 15, 30 and 50 min).

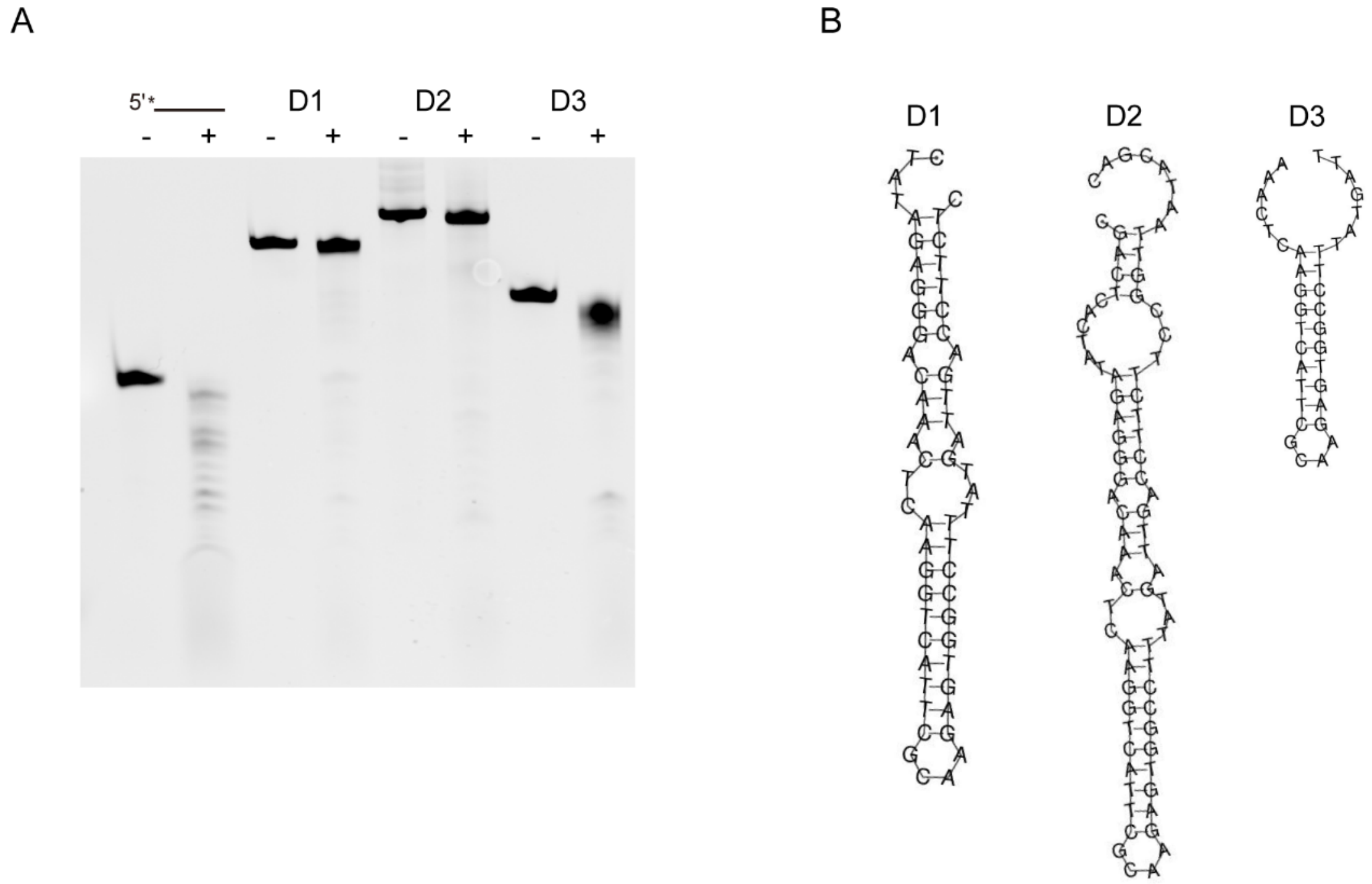

Supplementary Figure 5. Structure-specific 3′-5′ exonuclease activity of QatD. **A)** Cleavage assays with 5′-FAM-labeled oligonucleotide substrates. Each substrate (D1, D2, D3) was incubated with (+) or without (-) 0.5 μM QatD at a DNA: protein molar ratio of 1:5 in the presence of 10 mM Mg^{2+} for 60 min at 37 °C. Reactions were resolved by denaturing PAGE. Progressive laddering indicates 3′-5′ exonuclease degradation. **B)** Predicted secondary structures of the oligonucleotide substrates (D1, D2, D3) used in the assays. QatD exhibited consistent exonuclease activity across these substrates, indicating structure-specific rather than sequence-specific recognition.

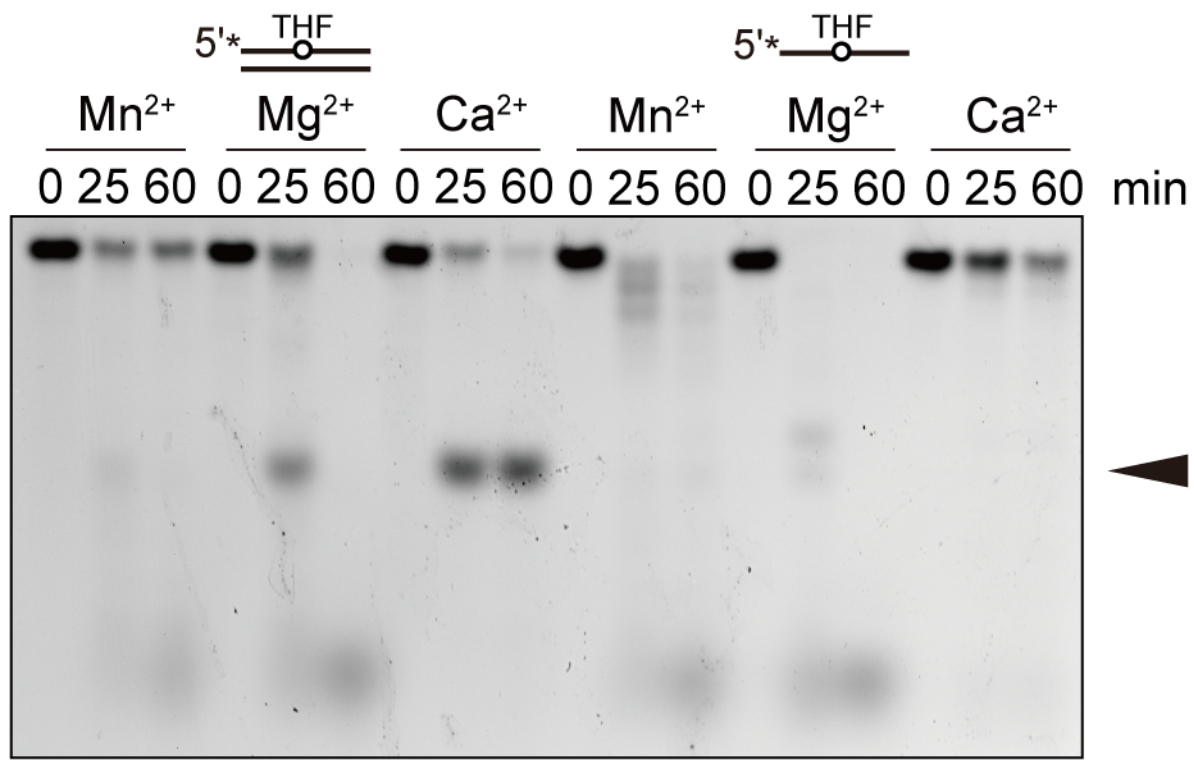

Supplementary Figure 6. Nuclease activity of excess QatD toward dsTHF and ssTHF substrates. Urea-PAGE analysis of 5'-FAM labeled dsTHF and ssTHF degradation by QatD at a 30:1 protein-to-DNA molar ratio. In the presence of Mg^{2+}, an intermediate stalled band (black arrowhead, representing exonucleolytic cleavage up to the THF site) appeared at 25 min but disappeared by 60 min. In contrast, this stalled band persisted significantly at 60 min in the presence of Ca^{2+}, indicating that Ca^{2+} specifically mediates the AP endonuclease activity of QatD. The 12 bp product band is indicated by a black triangle.

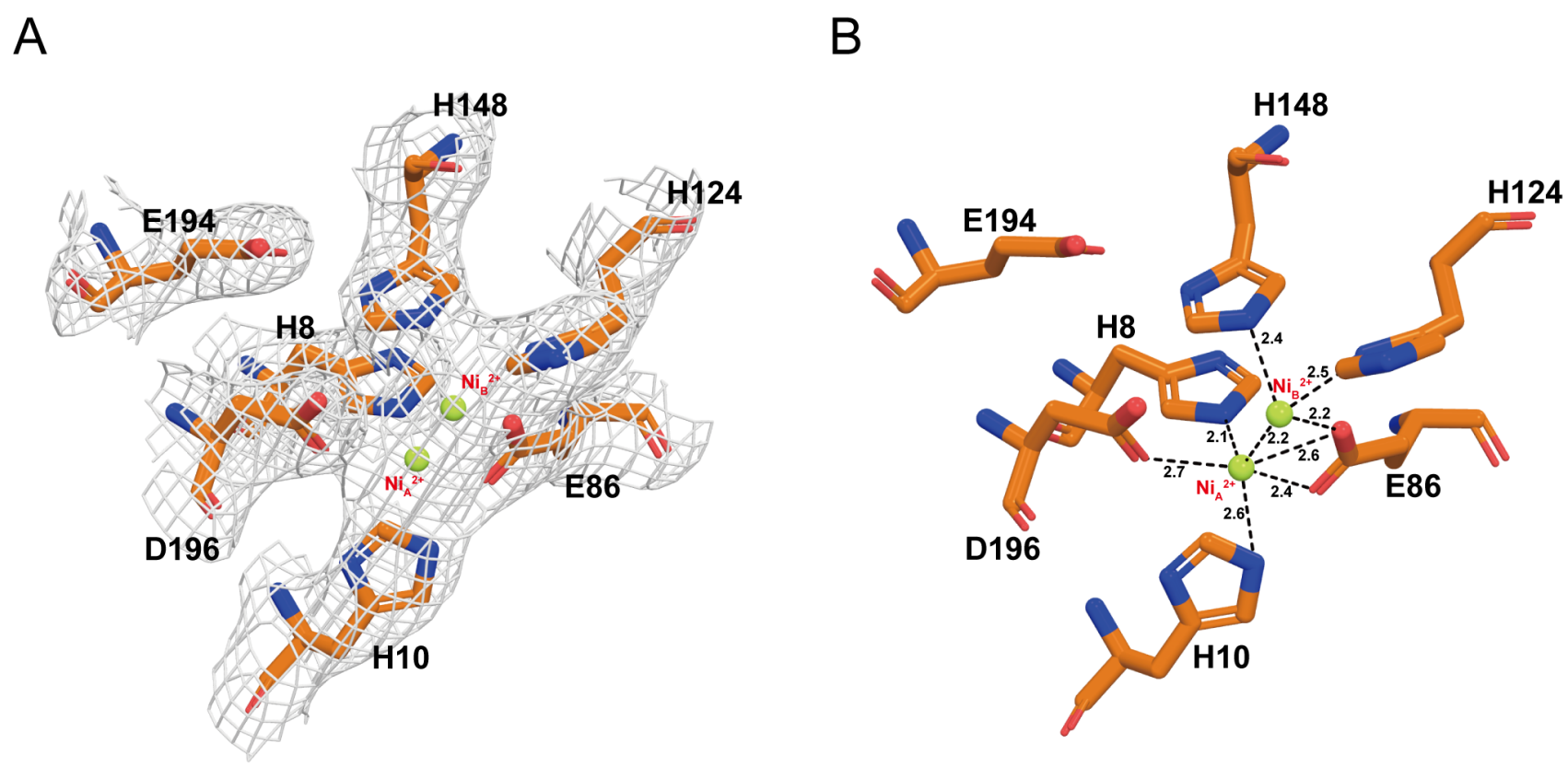

Supplementary Figure 7. Electron density map and coordination geometry of the di-nickel active site. **A)** Electron density map (grey mesh) of the di-nickel active site. The simulated-annealing *2mFo-DFc* electron density map was contoured at 2.0 σ. The two Ni^{2+} ions, denoted as Ni_A^{2+} and Ni_B^{2+}, are shown as limon spheres. Key coordinating amino acid residues are displayed as cyan sticks, with nitrogen and oxygen atoms colored blue and red, respectively. **B)** Detailed view of the metal coordination network. Black dashed lines represent the metal coordination bonds between the Ni^{2+} ions and the surrounding residues, with corresponding distances indicated in Ångströms (Å).

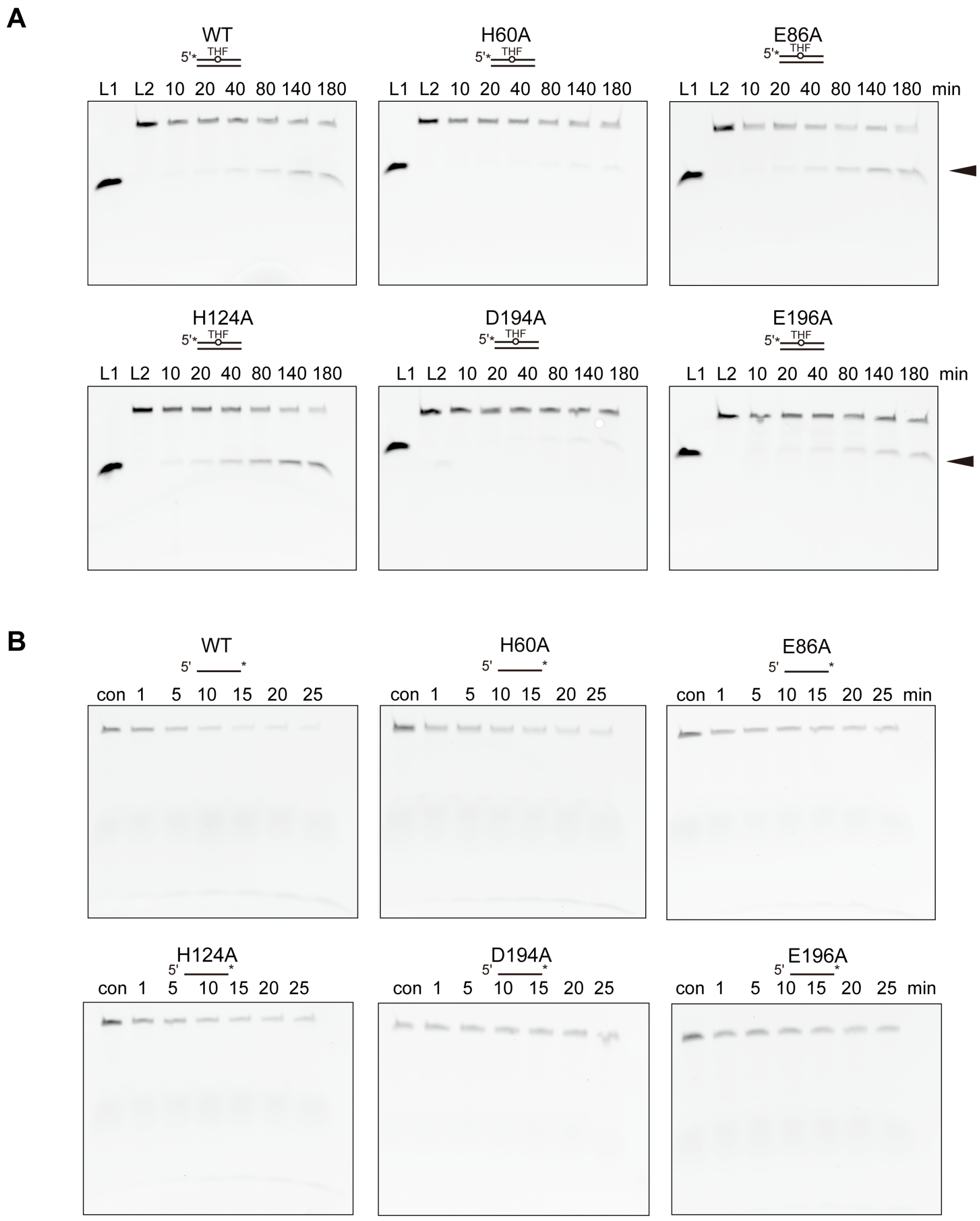

Supplementary Figure 8. Nuclease activity analysis of wild-type QatD and its mutants. **A)** Activity of 0.5 µM QatD and its mutants against 0.1 µM dsTHF substrates. The degradation of 5'-FAM labeled dsTHF by each protein was monitored over time (10–180 min) via Urea-PAGE in the presence of 5 mM Ca^{2+}. L1: 12 bp marker; L2: no-protein control. The 12 bp product band is indicated by a black triangle, all assays were performed in triplicate with reproducible results. **B)** Exonuclease activity of 0.5 µM QatD and its mutants against 0.1 µM ssDNA in the presence of 10 mM Mg^{2+}. Urea-PAGE analysis of 3'-FAM labeled ssDNA degradation by each protein. The con represents no-protein control, all assays were performed in triplicate with reproducible results.

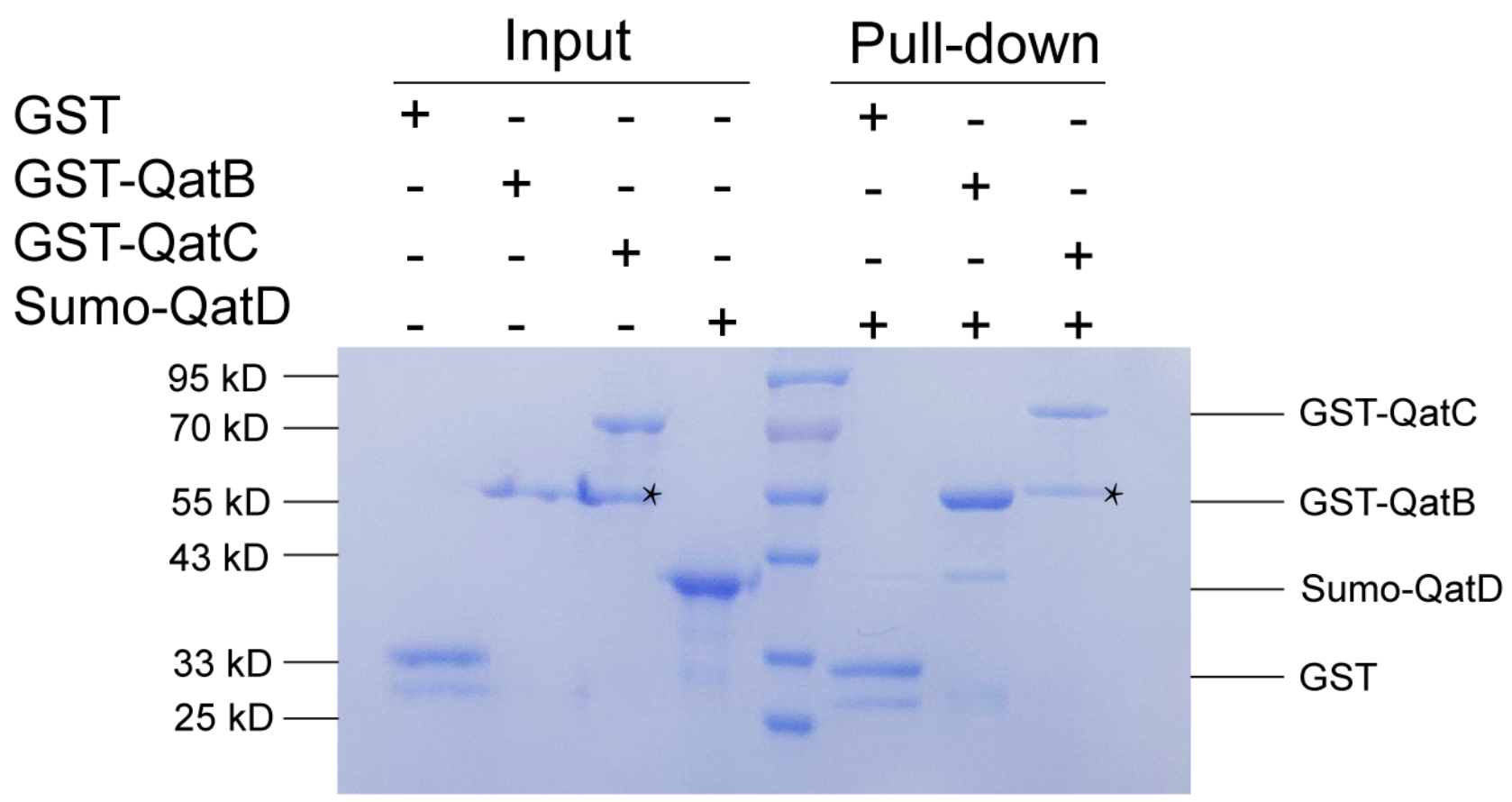

Supplementary Figure 9. GST Pull-down assay reveals the interaction between QatD and QatB. The minor band marked with a black star in the GST-QatC lane corresponds to a co-eluted host purification contaminant. Co-precipitation of Sumo-QatD was strictly exclusive to the GST-QatB bait, with no detectable background in the GST-only or GST-QatC control channels, indicating a specific physical interaction.
